# Tigecycline causes loss of cell viability mediated by mitochondrial OXPHOS and RAC1 in hepatocellular carcinoma cells

**DOI:** 10.1186/s12967-023-04615-4

**Published:** 2023-12-02

**Authors:** Dominik T. Koch, Haochen Yu, Iris Beirith, Malte Schirren, Moritz Drefs, Yunfei Liu, Mathilda Knoblauch, Dionysios Koliogiannis, Weiwei Sheng, Enrico N. De Toni, Alexandr V. Bazhin, Bernhard W. Renz, Markus O. Guba, Jens Werner, Matthias Ilmer

**Affiliations:** 1 Department of General, Visceral and Transplantation Surgery, LMU University Hospital, Ludwig-Maximilians-University (LMU) Munich, Marchioninistr. 15, 81377 Munich, Germany; 2 German Cancer Consortium (DKTK), DKTK Partner Site Munich, Munich, Germany; 3 Transplantation Center Munich, LMU University Hospital, Ludwig-Maximilians-University (LMU) Munich, Munich, Germany; 4 Liver Center Munich, Ludwig-Maximilians-University Munich, Munich, Germany; 5 Bavarian Cancer Research Center (BZKF), Munich, Germany; 6 Department of Internal Medicine II, LMU University Hospital, Ludwig-Maximilians-University (LMU) Munich, Munich, Germany; 7 Department of Gastrointestinal Surgery, The First Hospital, China Medical University, Shenyang, 110001 China

**Keywords:** Tigecycline, HCC, Drug repurposing, Mitochondrial oxidative phosphorylation, RAC1, ROS

## Abstract

**Background:**

Despite recent advances in locoregional, systemic, and novel checkpoint inhibitor treatment, hepatocellular carcinoma (HCC) is still associated with poor prognosis. The feasibility of potentially curative liver resection (LR) and transplantation (LT) is limited by the underlying liver disease and a shortage of organ donors. Especially after LR, high recurrence rates present a problem and circulating tumor cells are a major cause of extrahepatic recurrence. Tigecycline, a commonly used glycylcycline antibiotic, has been shown to have antitumorigenic effects and could be used as a perioperative and adjuvant therapeutic strategy to target circulating tumor cells. We aimed to investigate the effect of tigecycline on HCC cell lines and its mechanisms of action.

**Methods:**

Huh7, HepG2, Hep3B, and immortalized hepatocytes underwent incubation with clinically relevant tigecycline concentrations, and the influence on proliferation, migration, and invasion was assessed in two- and three-dimensional in vitro assays, respectively. Bioinformatic analysis was used to identify specific targets of tigecycline. The expression of RAC1 was detected using western blot, RT-PCR and RNA sequencing. ELISA and flow cytometry were utilized to measure reactive oxygen species (ROS) generation upon tigecycline treatment and flow cytometry to detect alterations in cell cycle. Changes in mitochondrial function were detected via seahorse analysis. RNA sequencing was performed to examine involved pathways.

**Results:**

Tigecycline treatment resulted in a significant reduction of mitochondrial function with concomitantly preserved mitochondrial size, which preceded the observed decrease in HCC cell viability. The sensitivity of HCC cells to tigecycline treatment was higher than that of immortalized non-cancerous THLE-2 hepatocytes. Tigecycline inhibited both migratory and invasive properties. Tigecycline application led to an increase of detected ROS and an S-phase cell cycle arrest. Bioinformatic analysis identified RAC1 as a likely target for tigecycline and the expression of this molecule was increased in HCC cells as a result of tigecycline treatment.

**Conclusion:**

Our study provides evidence for the antiproliferative effect of tigecycline in HCC. We show for the first time that this effect, likely to be mediated by reduced mitochondrial function, is associated with increased expression of RAC1. The reported effects of tigecycline with clinically relevant and achievable doses on HCC cells lay the groundwork for a conceivable use of this agent in cancer treatment.

**Supplementary Information:**

The online version contains supplementary material available at 10.1186/s12967-023-04615-4.

## Background

Hepatocellular carcinoma (HCC) is the most common primary tumor of the liver and accounts for more than 90% of all malignant primary liver tumors. Current data show that HCC is the sixth most frequent cancer entity worldwide and the third leading cancer-related cause of death. In male patients, in particular, the cancer mortality rate is the second highest [1–3]. The 5-year survival of HCC is 18% [4].

There are several treatment options for HCC including surgical resection, liver transplantation, chemoembolization, local ablative procedures, systemic chemotherapy, and checkpoint inhibitor therapy [6]. Liver transplantation (LT) is preferred for the treatment of HCC patients with underlying hepatic cirrhosis as it achieves 5-year recurrence rates of 15%, whereas recurrence rates after liver resection (LR) can be as high as 70% or more [7, 8]. However, LT is limited especially in countries with a lower donor organ pool. Here, patients who meet the Milan criteria are almost the only ones that can be transplanted [9].

Due to these limitations, resection must be performed despite high recurrence rates. Finding ways to lower recurrence rates after resection is crucial. Therefore, it is important to develop new perioperative and adjuvant therapeutic strategies. In this regard, drug repurposing is an attractive and feasible option. To date, adjuvant therapy options have not been fully established. The first-line protein kinase inhibitor Sorafenib, which is indicated for patients in BCLC advanced stage (C) with preserved liver function, ECOG-PS score of 1 or 2, and macrovascular invasion or extrahepatic spread [10], did not show any benefit when administered in an adjuvant setting [11].

Apart from new immunotherapies that are currently being tested [12], we here suggest the repurposing of the antibiotic tigecycline as a novel and innovative adjuvant and even perioperative treatment option. Given the favorable side effects of tigecycline, we may even suggest perioperative administration to target circulating tumor cells, which are a major cause of extrahepatic recurrence after LT and LR. Tigecycline could be used as a standard perioperative antibiotic and antitumor agent at the same time.

For many years, antibiotics have been used to treat bacterial infections. However, over the years in clinical practice, several antibiotics have been found to have an inhibitory effect on cell proliferation. In recent years, more and more basic science studies have investigated the antitumor effects of antibiotics.

Tigecycline, a glycylcycline antibiotic drug, is in use to treat several bacterial infections. It was originally developed to address rising rates of antibiotic resistance in Staphylococcus aureus, Acinetobacter baumannii, and Escherichia coli [13] and got fast-track approval from the FDA on June 17th, 2005 [14]. This broad-spectrum antibiotic inhibits mitochondrial protein synthesis. It exhibits its antimicrobial effect by binding to the 30S ribosomal subunit of bacteria, which interrupts the interaction of aminoacyl-tRNA with the ribosomal A site [15].

Tigecycline has been shown to have anticancer effects in studies of human acute myeloid leukemia, primarily by inhibiting mitochondrial translation [16]. Since then, an increasing number of anticancer studies on tigecycline have been published. Tigecycline has shown anticancer effects in various solid tumors, including gastric cancer [17], triple-negative breast cancer [18], cervical cancer [19], lung cancer [20], oral squamous cell carcinoma [21], and glioma [22], among others.

Whether tigecycline has a selective therapeutic effect on HCC without affecting normal hepatocytes remains unclear to date. To address this, our study explores the functional and mechanistic effects of tigecycline on HCC for the first time.

## Methods

### Cell culture

The human cell lines Huh7 and HepG2 were used and grown in RPMI 1640 medium (RPMI Gibco™; Thermo Fisher Scientific, Waltham, Massachusetts, USA), supplemented with 10% fetal bovine serum (FBS), in a humidified incubator with 5% CO_2_ at 37 °C. The human cell line Hep3B was grown in Minimum Essential Medium Eagle (Sigma-Aldrich, St. Louis, Missouri, USA), supplemented with 10% FBS, in a humidified incubator with 5% CO_2_ at 37 °C.

THLE-2 human normal liver epithelial cell line was purchased from the American Type Culture Collection (ATCC) and cultured in BEGM (Bronchial Epithelial Cell Growth Medium BulletKit™; Lonza, Basel, Switzerland). The kit includes 500 ml basal medium and separate frozen additives from which we discarded the gentamycin/amphotericin (GA) and epinephrine and from which we added an extra 5 ng/ml EGF, 70 ng/ml phosphoethanolamine and 10% FBS. The cell culture flasks used for the THLE-2 cell line were precoated with a mixture of 0.01 mg/ml fibronectin, 0.03 mg/ml bovine collagen type I, and 0.01 mg/ml bovine serum albumin dissolved in BEGM medium.

### MTT

Cell viability was assessed using a 3-(4,5-Dimethylthiazol-2-yl)-2,5-Diphenyltetrazolium Bromide (MTT) assay (Thermo Fisher Scientific, Waltham, Massachusetts, USA). According to the manufacturer’s instructions, cells were plated at a density of 8 × 10^4^/well in 96‑well plates with 1.25 µM to 160 µM of tigecycline and cell viability was assessed at different time points. The absorbance of each well was measured at a wavelength of 570 nm with a background wavelength of 670 nm using a VersaMax microplate reader (Molecular devices Instruments, San Jose, California, USA). Empty wells served as blank controls. The cell viability was calculated as a comparative percentage of the values obtained from untreated cells. As an additional control we used the solvent DMSO, which was necessary for tigecycline, and we used constant amounts of DMSO with increasing concentrations of tigecycline to avoid confounding. The standard curves were obtained according to nonlinear regression in GraphPad Prism 7.04 software and then the half maximum inhibitory concentrations (IC_50_) of tigecycline on different cells were calculated.

### Crystal violet assay

Cells were plated at a density of 8 × 10^4^/well in 96‑well plates with 1.25 µM to 160 µM of tigecycline and cell viability was assessed at different time points. The cells were washed with phosphate buffered saline (PBS) to ensure that all medium was removed. 50 µl of 4% paraformaldehyde (PFA) were added and an incubation for 15 min at room temperature followed. PFA was discarded and plates were allowed to air dry for 15 to 20 min. Then, cells were stained with 50 µl of crystal violet (CV) solution for 15 min. After discarding the CV, the cells were washed with H_2_O and it was allowed to dry overnight at room temperature. The next day, the dye was dissolved in 50 µl of 33% acetic acid. The absorbance of the developed color was measured at a wavelength of 570 nm with a background wavelength of 670 nm using VersaMax microplate reader (Molecular devices Instruments, San Jose, California, USA). Empty wells served as blank controls. The cell viability was calculated as a comparative percentage to the values obtained from untreated cells. As an additional control we used the solvent DMSO, which was carrier solution for tigecycline.

### Cell viability assay

Cell viability was assessed by quantifying the amount of ATP using the CellTiter-Glo 2.0 Cell Viability Assay (Promega, Walldorf, Germany) according to the manufacturer’s instructions. The cells were seeded into 6-well plates (Corning, New York, USA) for a final confluency of 85%, followed by treatment. After trypsinization, 100 µl of the cell suspension were transferred into black 96-well f-bottom plates (Greiner Bio-One, Frickenhausen, Germany). Subsequently, 100 µl of CellTiter-Glo reagent was added to each well and the cells were incubated at room temperature in the dark for 5 min. The luminescence signal was quantified with FilterMax F3 microplate reader (Molecular Devices Instruments, San Jose, California, USA) and evaluated through SoftMax Pro 7.1.2 GxP (Molecular Devices Instruments, San Jose, California, USA).

### Sphere formation assay

The human cell lines Huh7 and HepG2 were cultured in suspension in sphere formation assay (SFA) medium which consisted of serum-free DMEM/F12 supplemented with B27 (1:50, Gibco™; Thermo Fisher Scientific, Waltham, Massachusetts, USA), 10 ng/ml epidermal growth factor (EGF) and 20 ng/ml basic fibroblast growth factor (bFGF) (ImmunoTools, Friesoythe, Germany) and 1% methylcellulose. To form HCC spheres, 1000 suspended cells were cultured per well in ultra-low attachment plates (Corning, New York, USA). After 8 days of treatment, the formed spheres were inspected under the microscope and pictures were taken with 40 × magnification. The number of clones and their size were then analyzed by ImageJ software (open-source software).

### Wound healing assay

Huh7 and HepG2 cells were seeded in 6-well plates. When the cells grew in a full monolayer, a wound was produced by a straight scratch across the cell monolayer using a 200 μl sterile tip. The cells were then washed gently with PBS and new serum-free medium was added. Pictures were taken immediately (0 h) as well as after 24 and 48 h in the same location and with the same magnification. The area of each wound was analyzed at different time points using ImageJ software (open-source software). The reduction of the wound area was then calculated and interpreted as the cell migration ratio. The migration ratio was defined as the reduction of wound area under treatment related to the reduction of wound area without treatment.

### Transwell assay

Matrigel (Matrigel GFR Basement Membrane Matrix, cat. no. 354230; Corning, New York, USA) and serum-free medium were dissolved at 4 °C at a ratio of 1:3, mixed thoroughly, and added to the upper chamber of an 8.0 µm pore size transwell plate (Corning, New York, USA). 40 µl were added to each chamber so that it fully covered the bottom of the chamber. Then it was incubated in a humidified incubator with 5% CO_2_ at 37 °C overnight to allow the matrigel to fully solidify. A total number of 100,000 cells in 300 µl serum-free medium were seeded into the upper chamber of the transwell plate and 600 µl complete medium was added to the lower compartment. The cells were incubated for 24 h in a humidified incubator with 5% CO_2_ at 37 °C. After incubation for 24 h, the upper chamber was wiped twice with cotton swabs. Next, the cells were fixed with 4% paraformaldehyde for 15 to 20 min and stained with 0.5% crystal violet for another 15 to 20 min at room temperature. The numbers of invading cells in three randomly selected fields were counted under an inverted light microscope (TE2000-U Inverted Microscope, 100 × magnification; Nikon, Tokyo, Japan).

### Detection of reactive oxygen species

#### Two methods were applied to examine changes in Reactive Oxygen Species (ROS)

Evaluation via luminescence: The level of ROS was quantified by luminescence measurement using the Ros-GLO™ H_2_O_2_ Assay (Promega, Walldorf, Germany) according to the manufacturer’s instructions. The cells were seeded into white 96-well plates with clear flat-bottom (Thermo Fisher Scientific, Waltham, Massachusetts, USA) for a final confluency of 85%, followed by treatment for 48 h. Five hours prior to measurement, 20 µl of freshly prepared H_2_O_2_ Substrate Solution diluted with the Substrate Dilution Buffer at a ratio of 1:80 was added into the wells containing 80 µl of corresponding media. This was followed by adding 100 µl ROS-Glo Detection Solution prepared with Recon Buffer and Luciferin, D-Cysteine 100x, and Signal Enhancer in a ratio of 100:1:1 and incubation at room temperature for 20 min. The luminescence signal was measured with the FilterMax™ F3 microplate reader (Molecular Devices Instruments, San Jose, California, USA) and evaluated through SoftMax® Pro 7.1.2 GxP (Molecular Devices Instruments, San Jose, California, USA).

Evaluation via flow cytometry: The cells were cultured in 6-well plates (Corning, New York, USA) and incubated for 48 h at 37 °C with a final confluency of 85%. This was followed by trypsinization and subsequent transfer into 5 ml round-bottom polystyrene test tubes (Corning, New York, USA) and centrifuged at 500 rpm for 5 min. The pellet was resuspended in DPBS, followed by repetition of the washing step, and the supernatant was discarded. Determination of ROS by flow cytometry was performed using the Total Reactive Oxygen Species (ROS) Assay Kit 520 nm (Invitrogen, Carlsbad, California, USA) according to the manufacturer's instructions. The cells were supplied with 500 µl of DPBS prior to measurement. The events were recorded in the FITC-A channel and gated using FlowJo v10 (BD Life Sciences, Ashland, California, USA).

### Measurements of mitochondrial respiration

To assess mitochondrial respiratory function, oxygen consumption rates (OCR) were analyzed using the Seahorse XFp Analyzer (Agilent Technologies, Santa Clara, California, USA). 12,000 cells were seeded in Seahorse 8-well mini-plates per well and cultured at 37 ℃ with 5% CO_2_ and treated with 10 µM to 160 µM of tigecycline for 48 h. After 48 h of treatment, media was replaced with Seahorse assay medium, cells were incubated at 37 ℃ without CO_2_ for 1 h and then 2 µM oligomycin and 0.5 µM rotenone/antimycin A were added sequentially to assess mitochondrial respiratory capacity. For short-term treatment of Huh7, HepG2, and THLE-2, cells were treated with tigecycline in Seahorse 8-well mini-plates for only 6 h. After 6 h of treatment, the medium was replaced with a seahorse analysis medium and then the subsequent measurements were performed. Also, mitochondrial basal respiration, ATP production, and proton leak were measured according to the manufacturer’s protocol.

### Measurement of mitochondrial mass

Cells were cultured as described before, followed by trypsinization, subsequent transfer into 5 ml round-bottom polystyrene test tubes (Falcon, Corning, Wiesbaden, Germany) and centrifuged at 500 rpm for 5 min. The pellet was resuspended in DPBS, followed by repetition of the washing step, and the supernatant was discarded. To measure mitochondrial mass, we used Nonyl-Acridine Orange (NAO) (Acridine Orange 10-Nonyl Bromide) (Invitrogen, Carlsbad, California, USA). The cell pellet was fixed by drop-wise addition of ice-cold 70% ethanol with simultaneous vortexing. Ethanol was removed through two wash steps with DPBS followed by staining with 100 nm NAO diluted in DPBS, with incubation at 4 °C for 10 min in the dark. The cells were supplied with 500 µl of DPBS prior to measurement (BD LSRFortessa, BD Biosciences, San Jose, CA, USA). The events were recorded in the FITC-A channel and gated using FlowJo v10 (BD Life Sciences, Ashland, California, USA).

### Western blot analysis

Huh7, HepG2, and THLE-2 cells were washed twice with cold PBS and harvested with protein lysis buffer including protease and phosphatase inhibitors (Roche, Basel, Switzerland). The protein concentrations were assessed using a BCA Protein Assay kit (Thermo Fisher Scientific, Waltham, Massachusetts, USA). Equal amounts of proteins (25 μg/lane) from each group were separated by sodium dodecyl sulfate polyacrylamide gel electrophoresis on 10% and 13% gels (Bio‑Rad Laboratories, Hercules, California, USA) and transferred to polyvinylidene difluoride membranes (Merck Group, Darmstadt, Germany). After blocking with 5% bovine serum albumin (BSA) for 1 h at room temperature, the membranes were incubated with specific primary antibodies at 4 °C overnight. Then, after washing three times with tris‑buffered saline containing Tween‑20 (TBST), the membranes were incubated with horseradish peroxidase‑conjugated secondary antibodies for 1 h at room temperature. Proteins were visualized by chemiluminescence using enhanced chemiluminescent substrate (Bio‑Rad Laboratories, Hercules, California, USA). Immunoreactive bands were examined using the ChemiDoc Imaging System (Bio‑Rad Laboratories, Hercules, California, USA). The following antibodies were used: Rabbit Rac1/Cdc42 antibody (cat. no. 4561, dilution 1:1000; Cell Signaling Technology, Danvers, Massachusetts, USA), Rabbit p44/42 MAPK (Erk1/2) (cat. no. 4695, dilution 1:1000; Cell Signaling Technology, Danvers, Massachusetts, USA), Rabbit Phospho-p44/42 MAPK (Erk1/2) (cat. no. 8544, dilution 1:1000; Cell Signaling Technology, Danvers, Massachusetts, USA), Mouse OXPHOS antibody (cat. no. ab110411, dilution 1:1500; Abcam, Cambridge, United Kingdom) and Rabbit GAPDH antibody (cat. no. sc-25778, dilution 1:5000; Santa Cruz Biotechnology, Texas, USA). GAPDH was used as an internal control for each membrane.

### Flow cytometric analysis

Flow cytometry and BrdU Flow kits (BD Pharmingen, San Diego, California, USA) were used to analyze the cell cycle changes and live/dead cell populations of the three used cell lines Huh7, HepG2, and THLE-2 after tigecycline treatment. All cells were seeded in 6-well plates with different numbers of cells depending on the morphological size of the cells to obtain a confluence of 80% before treatment. For cell cycle analysis, culturing was then started at the same time for all groups. The control group was left untreated for the whole time of the experiment, the 24-h group was treated in the last 24 h and the 48-h group was treated in the last 48 h of the experiment. All samples were harvested at the same time. Therefore, 20 µl of BrdU solution (1 mM BrdU in 1xDPBS) were added directly to 2 ml of culture medium and the cells were incubated for 1 h. Then, the cells were trypsinized from the wells and transferred into FACS tubes. They were centrifuged for 5 min at 200 to 300 G and the supernatant was discarded. The cells were resuspended in 100 µl of BD Cytofix/Cytoperm buffer and incubated for 30 min at room temperature. After washing with 1 ml 1xBD Perm/Wash buffer, the cells were resuspended in 100 µl BD Cytoperm Permeabilization Buffer Plus and incubated for 10 min on ice. The measurements were performed by flow cytometry immediately after staining the cells with fluorescent anti-BrdU and 7-AAD solution according to the manufacturer’s instructions for the kit.

For live/dead cell staining, cells were trypsinized from the wells and transferred into FACS tubes. The cells were washed twice with 1xDPBS with centrifugation for 5 min at 200 to 300 G, and stained with 7-AAD for 10 min in the dark.

### RNA isolation and real‑time PCR

The total RNA from Huh7, and HepG2 cells was extracted with the Qiagen total RNA extraction kit (Qiagen, Venlo, Netherlands) and reverse transcription was performed using the SuperScript™ IV VILO™ Master Mix kit (Thermo Fisher Scientific, Waltham, Massachusetts, USA). The cDNA concentration was measured with NanoDrop (Thermo Fisher Scientific, Waltham, Massachusetts, USA). The quantitative RT‑PCR was performed using QuantiNova™ SYBR Green PCR Kit (Qiagen, Venlo, Netherlands) in a 20 μl PCR mixture on a Bio‑Rad CFX96 Real‑Time PCR system (Bio‑Rad Laboratories, Hercules, California, USA) according to the manufacturer’s standard protocols. An initial step of 2 min at 95 °C was followed by 40 cycles of 5 s at 95 °C and 10 s at 60 °C. Each sample was performed in duplicate and negative control with sterile RNase-free H_2_O was. The housekeeping gene GAPDH was used to normalize the variation of cDNA. Three independent experiments were performed for each group. Relative gene expression was normalized to GAPDH and calculated using the 2‑ΔΔCq method.

### RNA sequencing and analysis

Cells were seeded into 6-well plates (Corning, New York, USA), grown for 48 h in the presence and absence of 10 µM tigecycline and harvested at a confluency of 85%. RNA was isolated using the RNeasy Mini Kit (Qiagen, Venlo, Netherlands). After RNA isolation, RNA integrity number (RIN) was measured using the Agilent 2100 Bioanalyzer system (Agilent Technologies, Santa Clara, California, USA). RNA with a RIN value > 7 was selected for mRNA sequencing (poly-A selected). The libraries were prepared using the Illumina stranded mRNAprep ligation kit (Illumina, San Diego, California, USA), following the manufacturer's instructions. After final quality control, the libraries were sequenced in a paired-end mode (2 × 100 bases) in the Novaseq6000 sequencer (Illumina, San Diego, California, USA) with a depth of ≥ 25 million reads per sample. The analysis of RNA sequencing data sets was conducted using the web-based platform, www.usegalaxy.eu.

### Bioinformatic analysis

The molecular structure of tigecycline was obtained from PubChem (https://pubchem.ncbi.nlm.nih.gov/). Information on a total of 6,876 HCC-related genes was retrieved from the GeneCards database using “hepatocellular carcinoma” as a keyword. According to a GeneCards Inferred Functionality Score (GIFtS) greater than 20, we selected the top 5.4% of genes which comprised a total of 376 genes. Similarly, a search in the Disgenet database (https://www.disgenet.org/) using “hepatocellular carcinoma” as a keyword comprised 96 relevant genes for HCC. Excluding the 43 genes contained in both databases, we obtained a total of 429 potential genes related to HCC. Potential target proteins for tigecycline and their corresponding genes were obtained from the PharmMapper database (http://www.lilab-ecust.cn/pharmmapper/) and the Comparative Toxicogenomics Database (CTD, http://ctdbase.org/). Hereby, we got a total of 34 potential target genes. These 34 potential target genes and the 429 relevant genes were intersected and we got 11 genes that are both potential targets of tigecycline and related to HCC. Furthermore, survival data for the 11 potential genes was retrieved from the Kaplan–Meier plotter (https://kmplot.com/analysis/) and data for differential gene expression were obtained from GEPIA (http://gepia.cancer-pku.cn/) and The Human Protein Atlas (https://www.proteinatlas.org/). STRING 11.5 was used to analyze the correlation of the 11 genes. KEGG (Kyoto Encyclopedia of Genes and Genomes) enrichment analysis was performed to determine the pathways significantly associated with the 11 potential target genes. Functional enrichment analysis of the 11 potential target genes was performed by the R programming language.

### Statistical analysis

All experiments were independently performed at least three times. The mean standard deviation (SD) was determined for each group. Statistical analyses were performed using one/two‑way analysis of variance (ANOVA) for multiple group comparisons or student's t‑test for individual comparisons. Statistical significance was considered at p < 0.05. In all statistical graphs, bar graphs represent the mean ± SD. *p < 0.05, **p < 0.01, ***p < 0.005, ****p < 0.001, and ns means no significance compared with the control group.

## Results

### Tigecycline reveals antiproliferative effects on HCC cells

To investigate the effects of tigecycline on HCC, the human HCC cell lines Huh7 and HepG2 were used. The HCC cell lines were treated with increasing concentrations of tigecycline (1.25 µM to 160 µM) for 24 and 48 h and their viability was examined by 3-(4,5-Dimethylthiazol-2-yl)-2,5-Diphenyltetrazolium Bromide (MTT) assays.

**Fig. 1:**
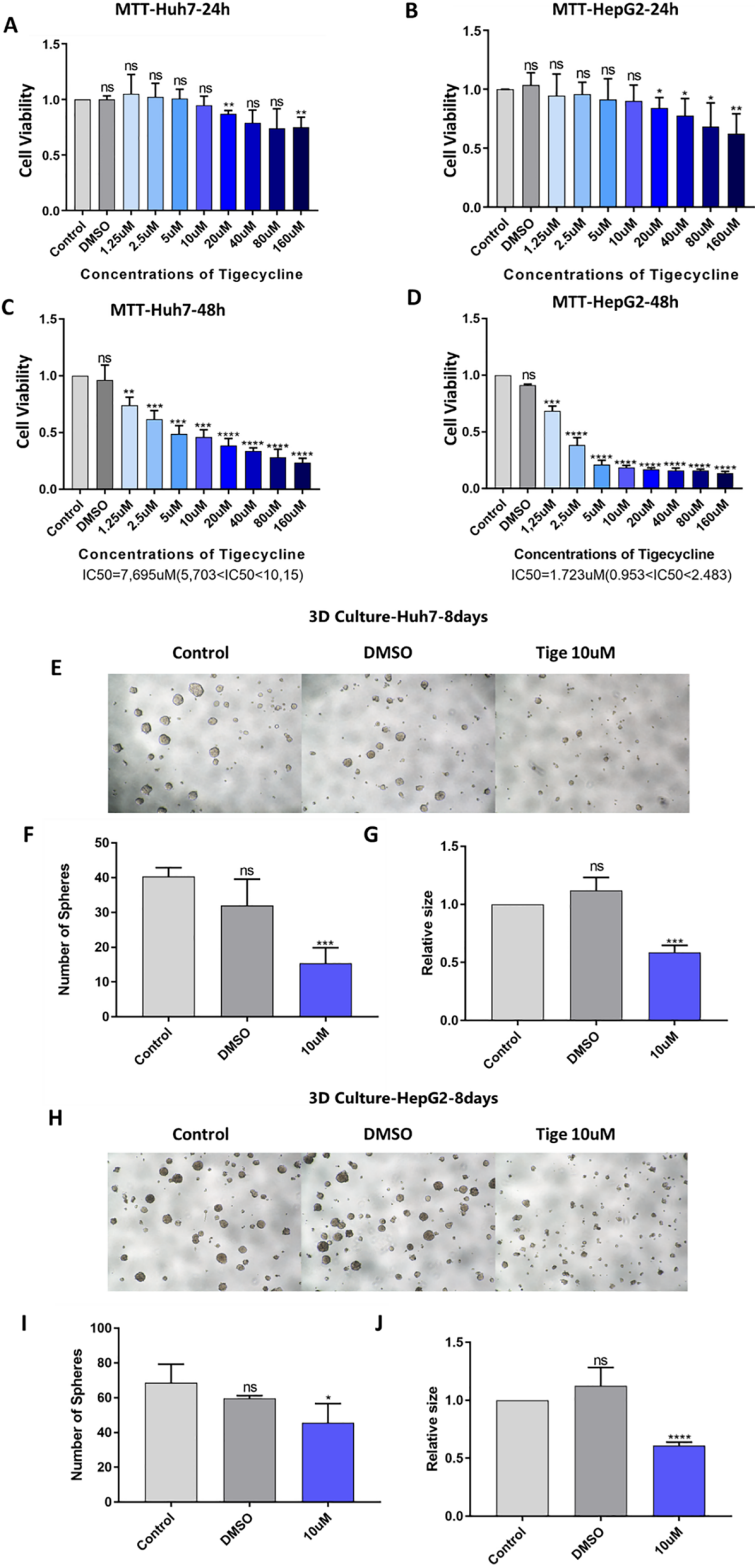
Tigecycline induced cytostatic effects on HCC cell lines Huh7 and HepG2 in two- and three-dimensional cell culture models. MTT based cell viability of Huh7 (**A**, **C**) and HepG2 (**B**, **D**) treated with increasing concentrations of tigecycline for 24 h (**A**, **B**) and 48 h (**C**, **D**); Microscope images of sphere formation of Huh7 (**E**) and HepG2 (**H**) cells after 8 days of incubation with 10 µM of tigecycline (magnification 100x); Corresponding statistical analysis of the number of spheres (**F**, **I**) and the size of spheres (**G**, **J**) for sphere formation of Huh7 (**F**, **G**) and HepG2 (**I**, **J**). Bar graphs represent the mean ± SD; *p < 0.05, **p < 0.01, ***p < 0.005, ****p < 0.001; ns = no significance compared with the control group (grey bar graphs). MTT, 3-(4,5-Dimethylthiazol-2-yl)-2,5-Diphenyltetrazolium Bromide; Tige, tigecycline; IC_50_, half maximal inhibitory concentration

Increasing concentrations of tigecycline did not influence the viability of Huh7 and HepG2 cells significantly after 24 h of treatment, except at very high and clinically irrelevant concentrations (Fig. 1 A and B). However, the cell viability of Huh7 and HepG2 decreased significantly in a dose-dependent manner after 48 h (Fig. 1C and D). We found that the half maximal inhibitory concentration (IC_50_) calculated for 48 h differed substantially between the two cell lines. HepG2 was more sensitive than Huh7 after 48 h of exposure to tigecycline (IC_50_ HepG2 1.723 µM vs. IC_50_ Huh7 7.695 µM).

To verify these results, we continued to test cell viability with crystal violet staining. Here, we achieved comparable results and comparable IC_50_ for both cell lines (data not shown). Due to the IC_50_ differences between HepG2 and Huh7 we added a third HCC cell line. We used the cell line Hep3B and got an IC_50_ of 2.673 µM based on MTT and calculated for 48 h (Additional file 1: Figure S1A and B). For all cell lines used in this project we additionally assessed cell viability with the CellTiter-Glo 2.0 Viability Assay that measures the amount of ATP present, indicating the amount of metabolically active cells. The assay confirmed the described effect of tigecycline on the viability of all HCC cell lines (Additional file 1: Figure S1C).

To elaborate whether these effects are due to cell death or proliferation inhibition, we carried out 7-AAD-staining and flow cytometry. In this regard, we did not detect any relevant increase in the number of dead cells after treatment with 10 and 20 µM tigecycline for 24 h and 48 h (Additional file 1: Figure S2 and S3).

To further investigate the effect of tigecycline on cancer stemness in vitro [23, 24], we performed sphere formation assays with Huh7 and HepG2 (Fig. 1E and H) and analyzed size and number of spheres after tigecycline treatment. We observed a significant reduction in number (Fig. 1F and I) and size (Fig. 1G and J) of spheres in both investigated HCC cell lines.

### Tigecycline inhibits migration and invasion of HCC cells

**Fig. 2:**
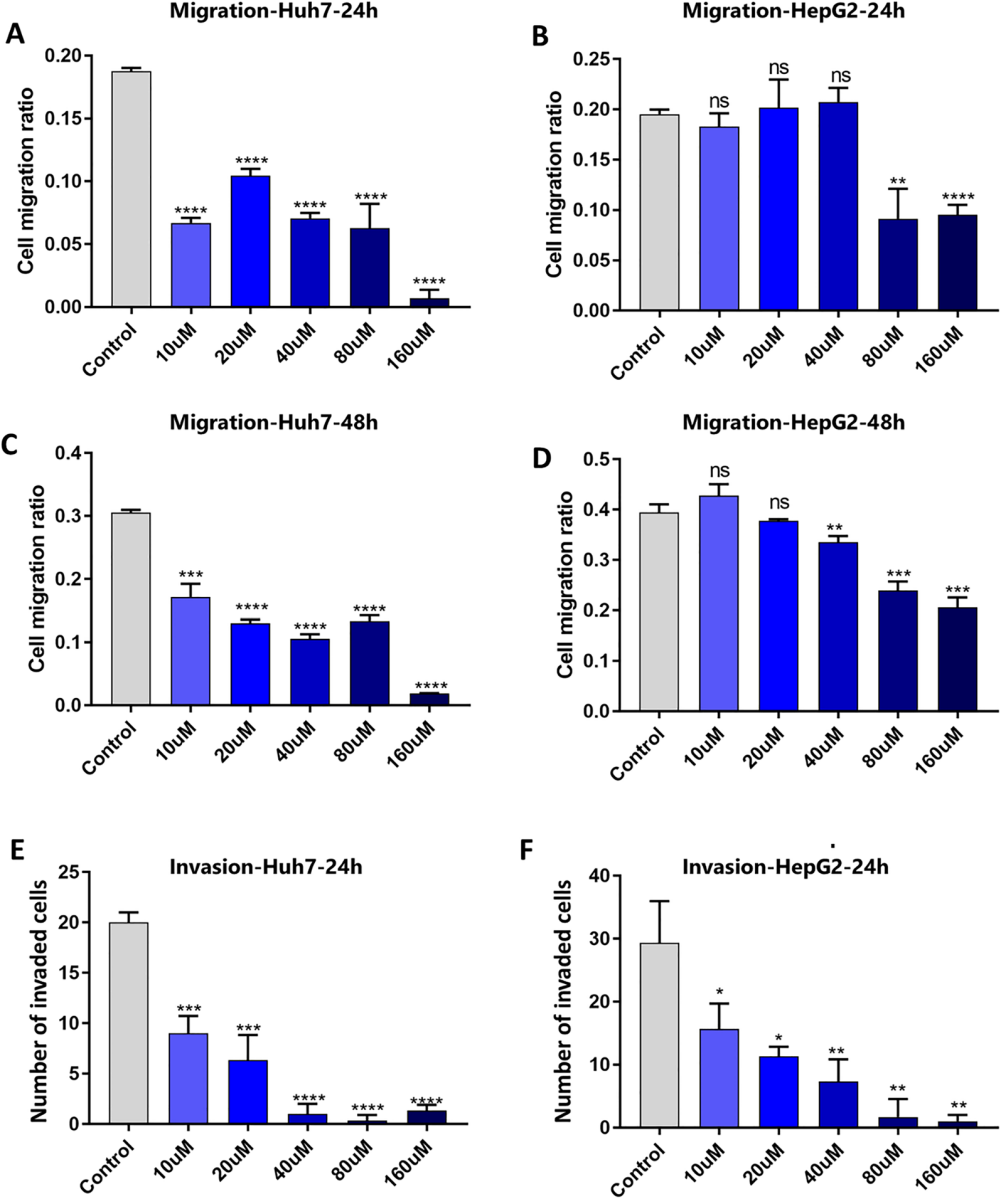
Tigecycline decreases migratory and invasive properties of HCC cells. Statistical analysis of wound healing assays with Huh7 and HepG2 after 24 h (**A**, **B**) and 48 h (**C**, **D**) of tigecycline treatment; Analysis of transwell invasion images with Huh7 and HepG2 after 24 h (**E**, **F**). Bar graphs represent the mean ± SD; *p < 0.05, **p < 0.01, ***p < 0.005, ****p < 0.001; ns = no significance compared with the control group (grey bar graphs). Migration ratio was defined as the reduction of wound area under treatment related to the reduction of wound area without treatment

After confirming the influence of tigecycline on the HCC cell viability, we further assessed tigecycline-related effects on HCC cell migration and invasion. Therefore, we performed wound healing assays to investigate the migration of HCC cells (Additional file 1: Figure S4A and C). After treating Huh7 cells with increasing concentrations of tigecycline (10 µM to 160 µM) for 24 h (Fig. 2A) and 48 h (Fig. 2C), we found that even low concentrations of tigecycline (starting at 10 µM) inhibited the migratory ability of Huh7 cells after 24 and 48 h. Migration of HepG2 was significantly inhibited at 80 µM or higher concentrations of tigecycline for 24 h (Fig. 2B) as well as 40 µM or higher concentrations for 48 h (Fig. 2D), respectively. Furthermore, transwell assays were used to test the invasion ability of Huh7 and HepG2 at increasing concentrations of tigecycline (Additional file 1: Figure S4B and D). As shown in Fig. 2 E, 10 µM of tigecycline significantly reduced the invasive capability of Huh7 cells after 24 h. Performing the same experiments with HepG2 cells, we observed comparable inhibition of invasion (Fig. 2F). The invasion capability was also significantly reduced at a concentration of 10 µM of tigecycline for 24 h.

### Tigecycline increases levels of reactive oxygen species in HCC cells

**Fig. 3:**
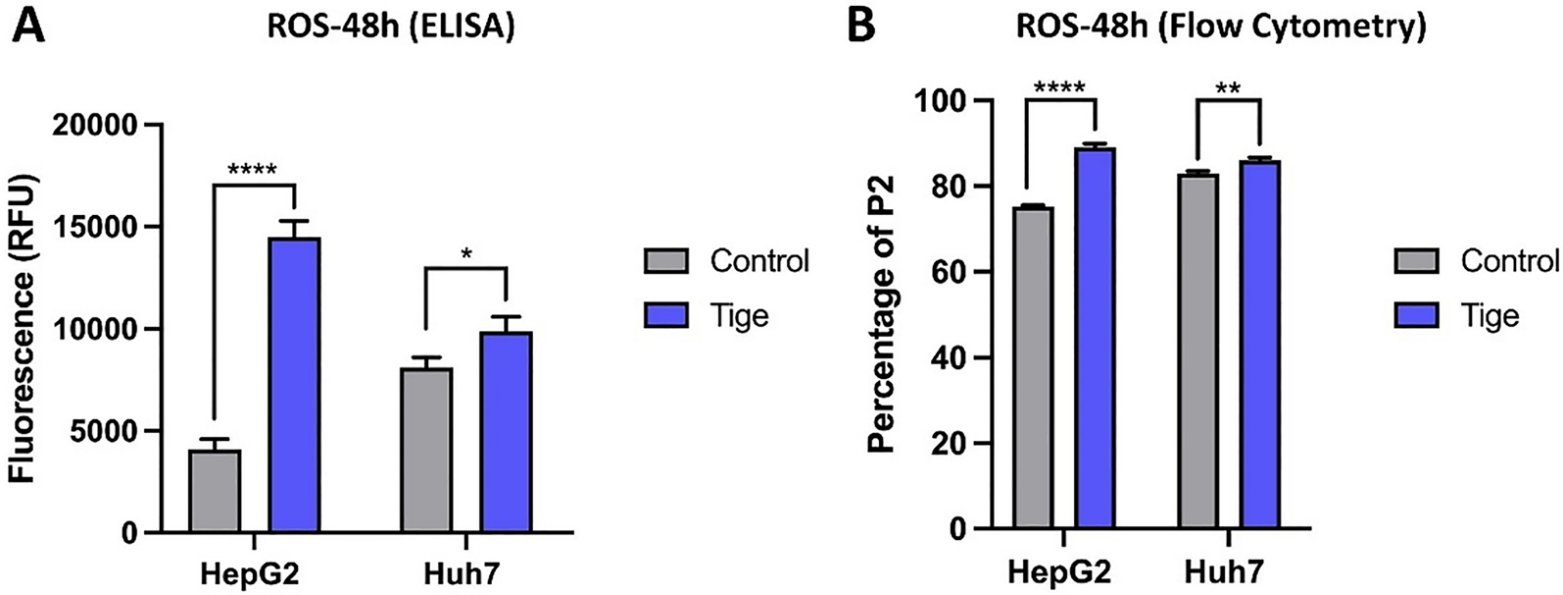
Treatment of HCC cells with tigecycline increases ROS levels. ELISA (**A**) and flow cytometry (**B**) based measurement of ROS levels in Huh7 and HepG2 after 48 h of treatment with 10 µM tigecycline. Bar graphs represent the mean ± SD; *p < 0.05, **p < 0.01, ***p < 0.005, ****p < 0.001; ns = no significance compared with the control group (grey bar graphs). *RFU* relative fluorescent units; Tige, tigecycline

Previous studies reported that tigecycline could promote the production of reactive oxygen species (ROS) [25]. To evaluate the effect of tigecycline on ROS in HCC, we performed ELISA- and flow cytometry-based assays in HepG2 and Huh7 cell lines. Here, we observed an increase in ROS levels after 48 h of treatment with tigecycline in both cell lines (Fig. 3A and B).

### Tigecycline impairs mitochondrial energy metabolism in HCC

**Fig. 4:**
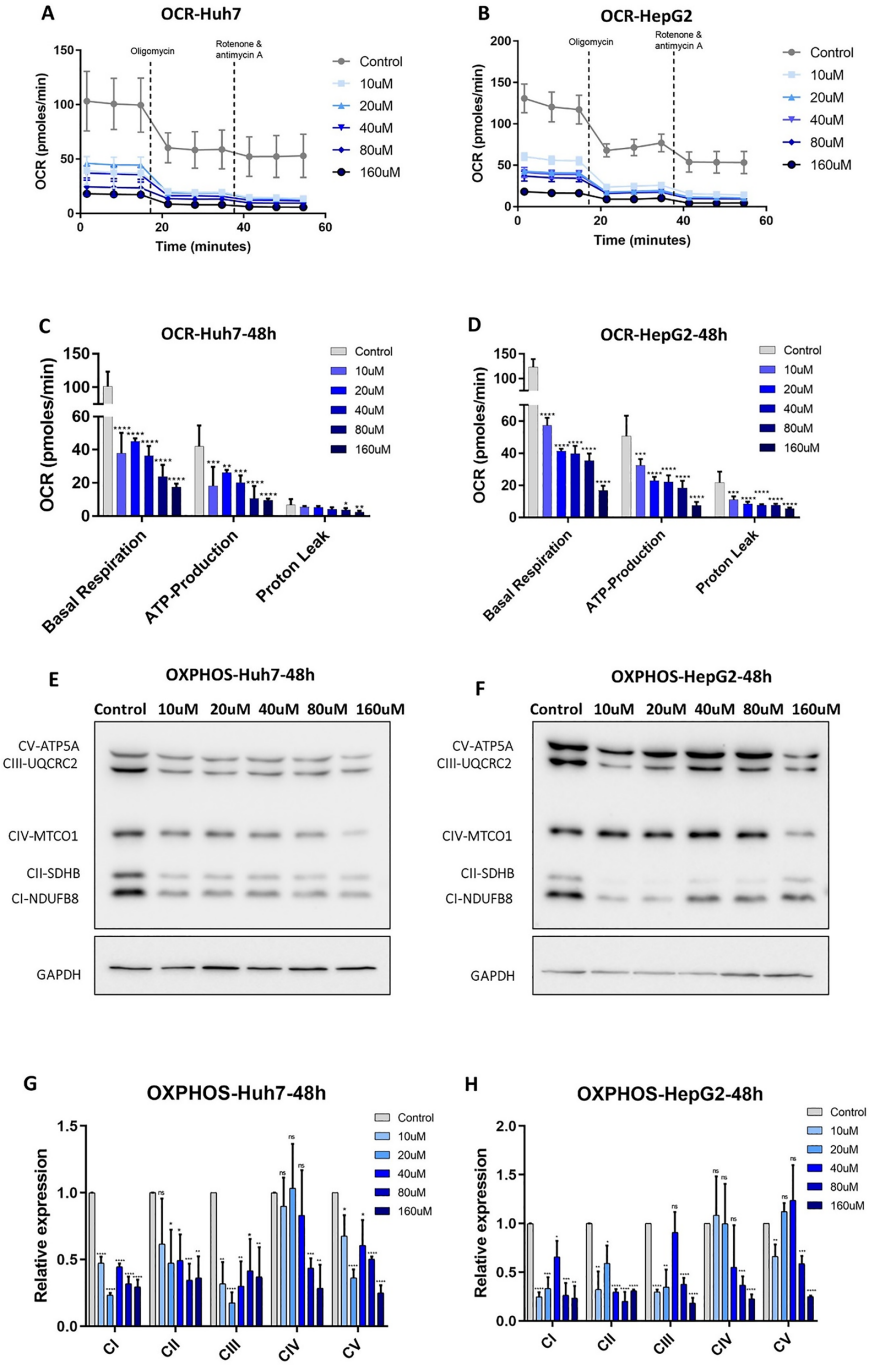
Mitochondrial OXPHOS and respiratory chain subunit expression after tigecycline treatment of HCC cells. OCR after 48 h of tigecycline treatment of Huh7 (**A**) and HepG2 (**B**); Statistical analysis of changes in basal respiration, ATP production and proton leak after treatment with different concentrations of tigecycline in Huh7 (**C**) and Hep G2 (**D**); Changes in the protein expression of respiratory chain subunits in Huh7 (**E**) and HepG2 (**F**) after tigecycline treatment; Relative protein expression of respiratory chain subunits after tigecycline treatment of Huh7 (**G**) and HepG2 (**H**) for 48 h. Bar graphs represent the mean ± SD; *p < 0.05, **p < 0.01, ***p < 0.005, ****p < 0.001; ns = no significance compared with the control group (grey bar graphs). OCR, oxygen consumption rate; *CI* Complex I; *CII* Complex II; *CIII* Complex III; *CIV* Complex IV; *CV* Complex V

We hypothesized that tigecycline may interfere with the metabolic cycle, in particular, with mitochondrial cellular OXPHOS, resulting in decreased viability as a consequence. Therefore, we analyzed oxygen consumption rates (OCR), that are representative of mitochondrial OXPHOS. OCR was significantly decreased after 48 h of treatment with 10 µM tigecycline in Huh7 and HepG2. The decrease was more pronounced with increasing tigecycline concentrations (10 µM to 160 µM) (Fig. 4A and B).

Further, we analyzed OCR basal respiration, ATP production, and proton leak. Here, we found a significant decrease after tigecycline treatment in a dose-dependent manner in both cell lines (Fig. 4C and D).

To assess how tigecycline treatment affects mitochondrial content, we analyzed the expression of subunits of the electron transport chain (ETC) (complexes I to IV) and of the ATP synthase (complex V) after tigecycline treatment by western blot. Tigecycline reduced the expression of all analyzed subunits of the ETC complexes and complex V in both Huh7 (Fig. 4E and G) and HepG2 (Fig. 4F and H). All analyzed subunits, including both nuclear (nuc) DNA- and mitochondrial (mt) DNA-encoded subunits (CIV-MTCO1), were reduced in a comparable manner.

### Tigecycline treatment increases mitochondrial mass in HCC cells

**Fig. 5:**
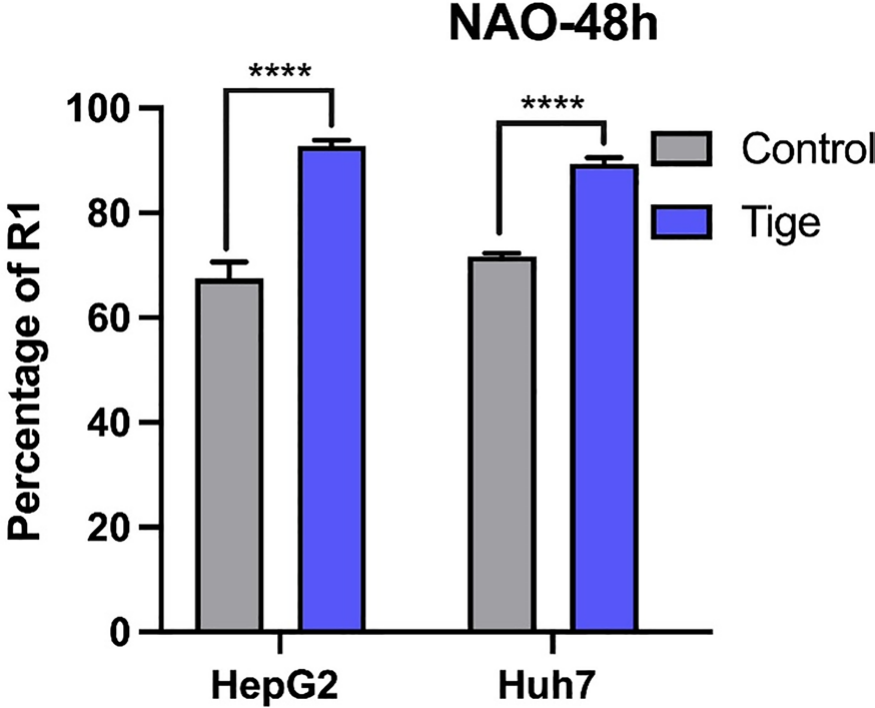
Mitochondrial mass increased after tigecycline treatment of HCC cells. Flow cytometry measurement of NAO after 48 h of tigecycline treatment in Huh7 and HepG2. Bar graphs represent the mean ± SD; *p < 0.05, **p < 0.01, ***p < 0.005, ****p < 0.001; ns = no significance compared with the control group (grey bar graphs). *NAO* Nonyl-Acridine Orange; *Tige* tigecycline

To examine whether the impairment of mitochondrial energy metabolism and the decrease of respiratory chain subunit protein levels is matched by a change in mitochondrial mass, we used Nonyl-Acridine Orange (NAO). We found that mitochondrial mass significantly increased after tigecycline treatment with 10 µM for 48 h in Huh7 and HepG2 cells (Fig. 5).

### Tigecycline exposure leads to cell cycle shift in HCC cells

**Fig. 6:**
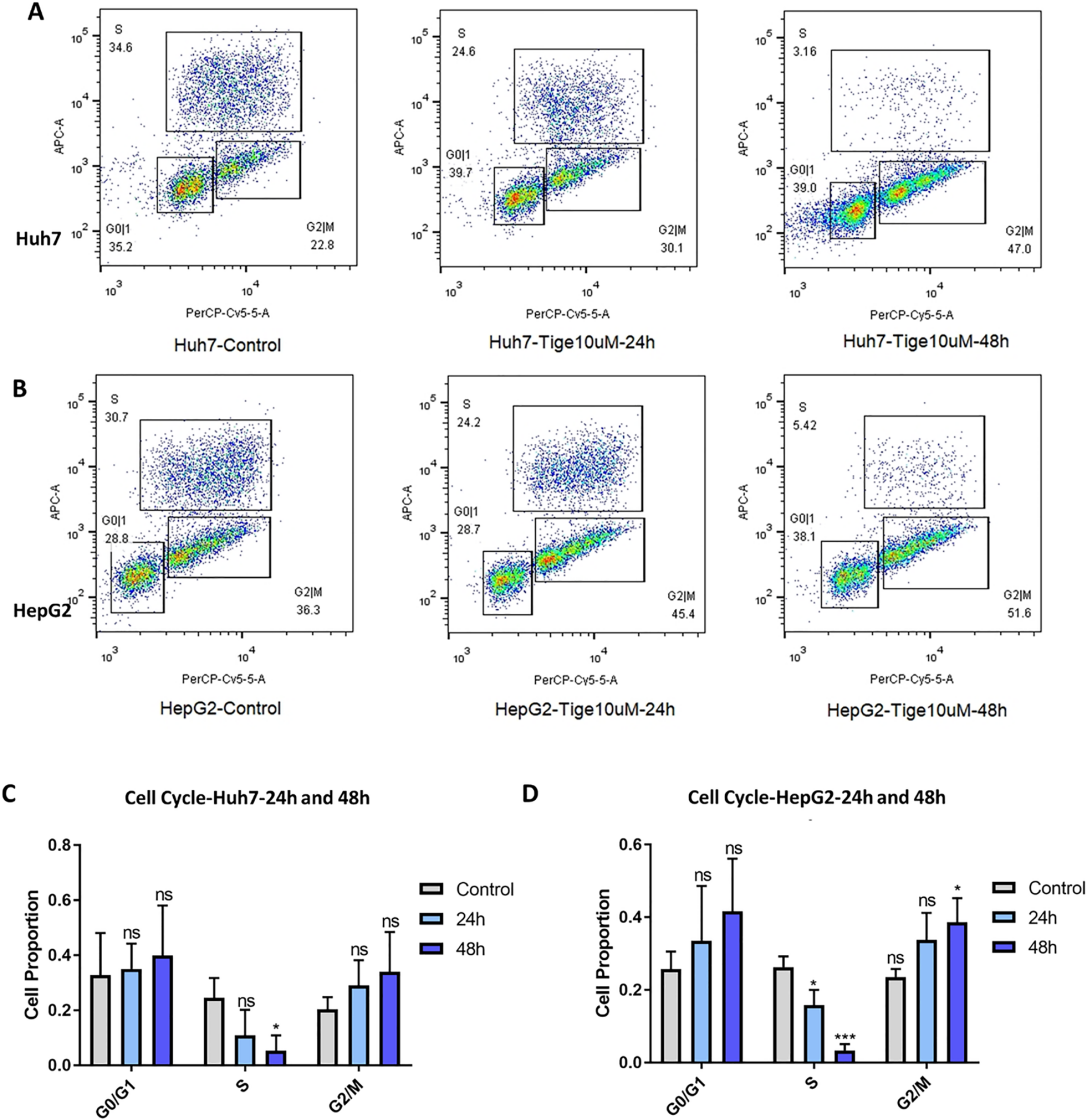
Cell cycle analysis of Huh7 and HepG2 cells after tigecycline treatment. Flow cytometric analysis of cell cycle in Huh7 (**A**) and HepG2 (**B**) after treatment with 10 µM tigecycline for 24 and 48 h, respectively; statistical analysis on the percentage of cells in different phases for Huh7 (**C**) and HepG2 (**D**). Bar graphs represent the mean ± SD; *p < 0.05, **p < 0.01, ***p < 0.005, ****p < 0.001; ns = no significance compared with the control group (grey bar graphs)

To further elaborate on the effect of tigecycline on HCC cells, we used BrdU-based flow cytometry assays to examine cell cycle changes after tigecycline treatment. In Huh7, 10 µM tigecycline reduced the percentage of cells in S-phase. This percentage further decreased with increasing duration of treatment with significant differences after 48 h. As the proportion of S-phase cells decreased, the proportion of G0/G1 and G2/M-phase cells increased (Fig. 6A and C). Similar results were found in HepG2 (Fig. 6B and D), where the decrease in S-phase already reached significance after 24 hourswith extension to 48 h.

### Malignant HCC cells tend to be more sensitive to Tigecycline than benign hepatocytes

**Fig. 7:**
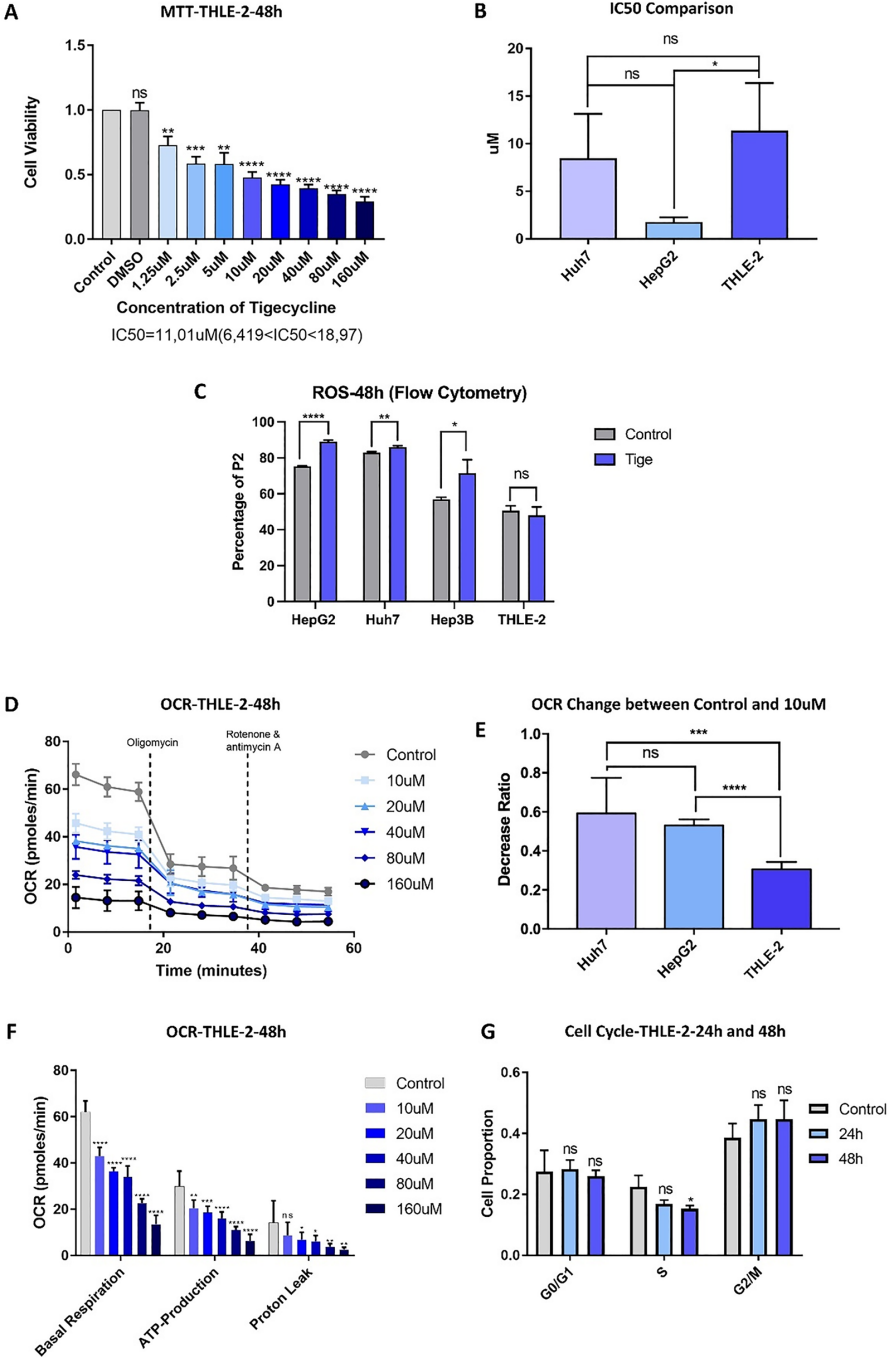
Tigecycline affects normal hepatocytes less compared to HCC cells. MTT based cell viability of THLE-2 treated with increasing concentrations of tigecycline for 48 h (**A**); Comparison of IC_50_ of Huh7, HepG2 and THLE-2 cells (**B**); Flow cytometry based measurement of ROS levels of THLE-2 compared to the HCC cells after 48 h of treatment with 10 µM of tigecycline (**C**); OCR after 48 h of tigecycline treatment of THLE-2 cells (**D**); Comparison of the relative reduction of OCR in basal respiration after treatment with 10 µM tigecycline for 48 h of Huh7, HepG2 and THLE-2 cells (**E**); Changes in basal respiration, ATP production and proton leak after treatment with different concentrations of tigecycline in THLE-2 cells (**F**); Statistical analysis on the percentage of cells in different phases for THLE-2 (**G**). Bar graphs represent the mean ± SD; *p < 0.05, **p < 0.01, ***p < 0.005, ****p < 0.001; ns = no significance compared with the control group (grey bar graphs). MTT, 3-(4,5-Dimethylthiazol-2-yl)-2,5-Diphenyltetrazolium Bromide; Tige, tigecycline; *OCR* oxygen consumption rate

To compare the above-reported effects with non-malignant hepatocytes, we used the immortalized normal epithelial liver cell line THLE-2. We simultaneously treated THLE-2 as well as HCC cell lines Huh7 and HepG2 with the same concentrations of tigecycline for 48 h and subsequently determined cell viability. In THLE-2 cells, cell viability decreased in a dose-dependent manner (Fig. 7A). However, the IC_50_ of THLE-2 (IC_50_ THLE-2 = 11.01 µM) was higher than that of Huh7 and HepG2 (IC_50_ Huh7 = 7.695 µM; IC_50_ HepG2 = 1.723 µM), indicating that both hepatocellular carcinoma cell lines were more susceptible to tigecycline treatment than THLE-2 (Fig. 7B). Furthermore, we repeated the experiments to detect alterations in ROS and OCR in THLE-2 cells after treatment with tigecycline. Here, we found no significant alteration in ROS after tigecycline treatment, in particular no increase in ROS as observed for the malignant HCC cells (Fig. 7C). For OCR, a decrease similar to Huh7 and HepG2 was observed (Fig. 7D). We recalculated the relative reduction of OCR after tigecycline treatment compared to the respective control groups for Huh7, HepG2, and THLE-2. We found that THLE-2 revealed statistically significant less decrease in basal respiratory OCR when compared to Huh7 and HepG2 at a concentration of 10 µM for 48 h. Tigecycline reduced the OCR by 59.18% in Huh7, 53.05% in HepG2, and only 30.58% in THLE-2 cells on average (Fig. 7E). Also, at a concentration of 20 µM tigecycline, the OCR was significantly less decreased in THLE-2 cells compared to Huh7 and HepG2 (Additional file 1: Figure S5A).

However, basal respiration, ATP production and proton leak decreased significantly in THLE-2 (Fig. 7F). Flow cytometry for cell cycle analysis in THLE-2 revealed that the S-phase of THLE-2 was significantly reduced after 48 h of treatment with 10 µM tigecycline, without significant changes to G0/G1 or G2/M phases (Fig. 7G, Additional file 1: Figure S5 B). In addition, we examined the expression of Complex I to V by western blot and found that 10 µM and 20 µM tigecycline decreased the expression of subunits of all respiratory chain complexes in THLE-2 cells (Additional file 1: Figure S5C and D).

### Tigecycline modifies OXPHOS prior to its effects on cell viability

Typically, in a rapidly proliferating human cell with a total cell cycle time of 24 h, the G1-phase should last about 11 h, S-phase 8 h, G2-phase 4 h, and M-phase 1 h [26]. Cell cycle fluctuations implicate metabolic and energetic changes. Given that background, we hypthesized that tigecycline may interfere with the metabolic cycle, in particular, with mitochondrial OXPHOS of cells, subsequently resulting in decreased cell viability. As shown above in Fig. 1, cell viability was significantly affected only after 48 h.of tigecycline exposure.

**Fig. 8:**
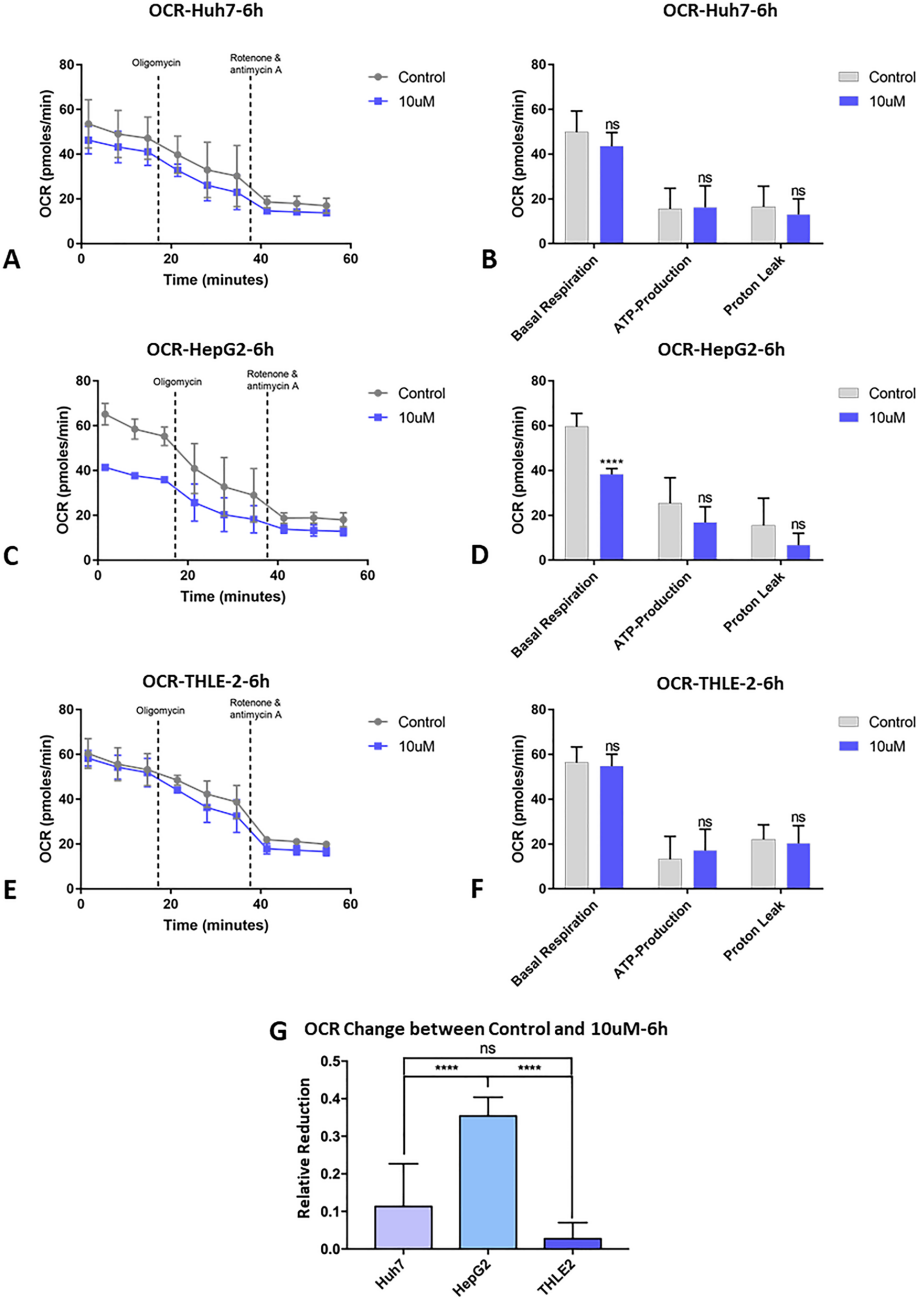
Alterations in mitochondrial OXPHOS after short-term tigecycline treatment of Huh7, HepG2, and THLE-2. OCR after 6 h of 10 µM tigecycline treatment of Huh7 (**A**), HepG2 (**C**) and THLE-2 (**E**); Changes in basal respiration, ATP production and proton leak after 10 µM tigecycline treatment of Huh7 (**B**), HepG2 (**D**) and THLE-2 (**F**); Comparison of the relative reduction of OCR decrease in basal respiration after 6 h of 10 µM tigecycline treatment compared to the respective control group of Huh7, HepG2 and THLE-2 cells (**G**). Bar graphs represent the mean ± SD; *p < 0.05, **p < 0.01, ***p < 0.005, ****p < 0.001; *ns*  no significance compared with the control group (grey bar graphs)

Therefore, we assessed mitochondrial OCR and basal respiration, ATP production, and proton leak in Huh7, HepG2 and THLE-2 with a shortened tigecycline exposure of 6 h. We found a statistically significant decrease of OCR and basal respiration in HepG2 (Fig. 8C and D). No significant decrease was found in in Huh7 (Fig. 8A and B). and (Fig. 8E and F) after 10 µM tigecycline treatment. The reduction of the basal respiratory OCR was 11.31% in Huh7, 35.39% in HepG2, and only 2.6% in THLE-2 cells on average (Fig. 8G).

### Bioinformatical analysis reveals RAC1 as a potential target for Tigecycline in HCC cells

To identify potential target proteins that mediate the effect of tigecycline in HCC cells, we conducted a detailed bioinformatical analysis using established databases. First, we obtained a total of 429 potential genes related to hepatocellular carcinoma from the GeneCards and DisNET databases. In addition, we used the two databases Pharmmaper and Comparative Toxicogenomics Database to identify potential target proteins of tigecycline and their corresponding genes. We obtained 34 potential target genes for tigecycline. These 34 potential targets were overlapped with 429 genes related to. This overlap resulted in 11 genes that are relevant in HCC and concomitantly code for potential targets of tigecycline (Additional file 1: Figure S6A). After differential expression analysis and survival prognosis analysis of these 11 genes using data from The Cancer Genome Atlas Program (TCGA), we found that survival only significantly differed for high and low expression of RAC1(Additional file 1: Figure S6B–F). Therefore, we postulated that RAC1 might play a major role in mediating the observed effects of tigecycline in HCC.

### Tigecycline treatment alters RAC1 RNA and protein expression in HCC cells

**Fig. 9:**
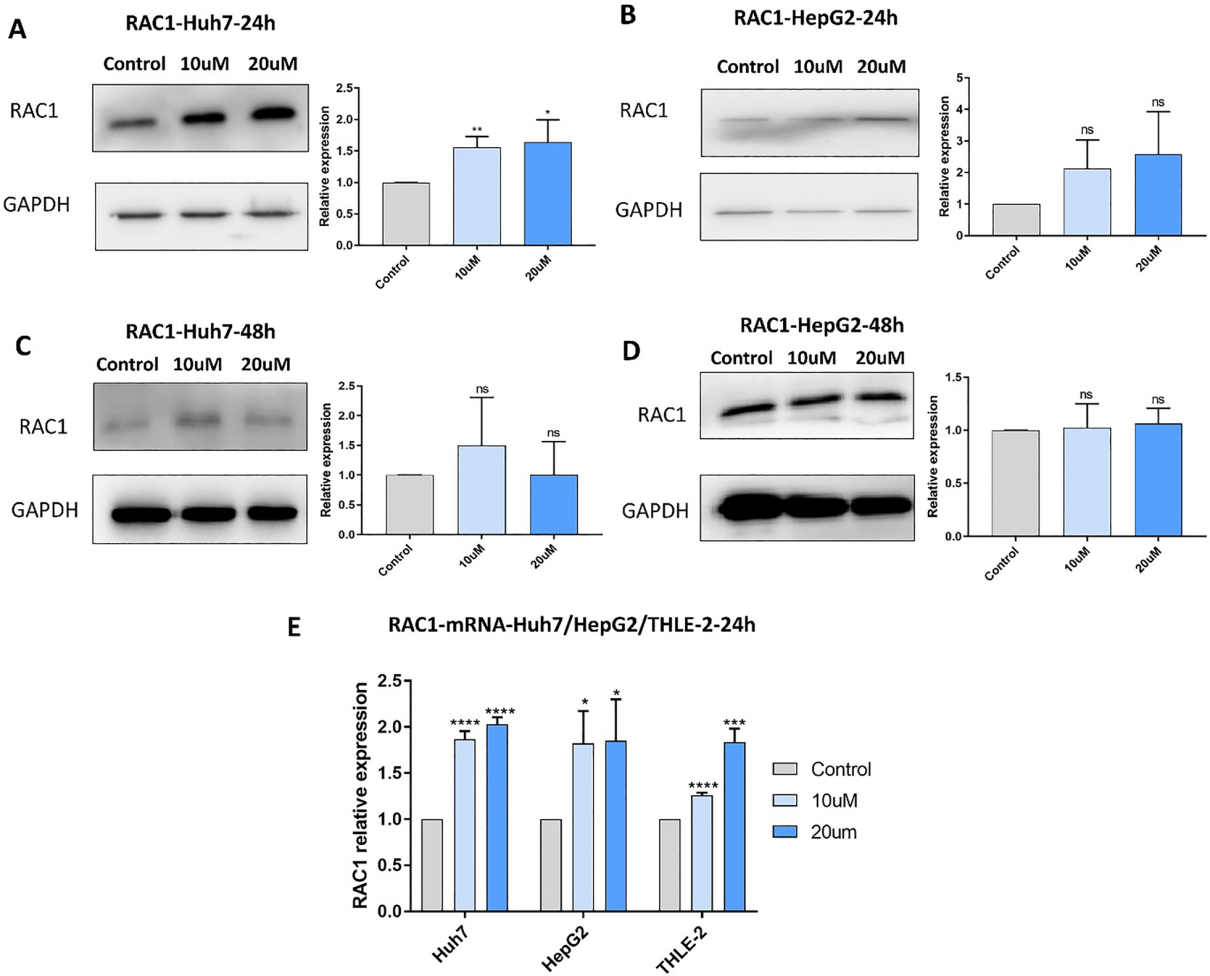
RAC1 protein and mRNA expression increases in HCC cells after treatment with tigecycline. RAC1 protein expression in Huh7 (**A**, **C**) and HepG2 (**B**, **D**) after treatment with 10 µM (light blue bar graphs) and 20 µM (dark blue bar graphs) tigecycline for 24 h (**A**, **B**) and 48 h (**C**, **D**); **E** RAC1 mRNA expression in Huh7, HepG2, and THLE-2 after treatment with 10 µM (light blue bar graphs) and 20 µM (dark blue bar graphs) tigecycline for 24 h. Bar graphs represent the mean ± SD; *p < 0.05, **p < 0.01, ***p < 0.005, ****p < 0.001; ns = no significance compared with the control group (grey bar graphs)

To assess the effects of tigecycline, we first detected protein expression of RAC1 by western blot after treatment with tigecycline (10 µM and 20 µM for 24 h) in Huh7. After tigecycline exposure, the protein expression of RAC1 increased significantly in a dose-dependent manner (Fig. 9A). In HepG2 the same tendency was observed (Fig. 9B). These effects were not obvious anymore after 48 h of treatment (Fig. 9C and D).

In contrast to Huh7 and HepG2, we did not obtain similar western blot results for THLE-2 (Additional file 1: Figure S6G). There was no measurable protein expression consistent with ourprevious bioinformatic analysis.

To detect mRNA expression of RAC1 after tigecycline treatment, we carried out RT-PCR for all three cell lines. In contrast to protein expression, we found RAC1 mRNA expression in all three cell lines. Both 10 µM and 20 µM of tigecycline promoted significantly higher RAC1 mRNA expression in all investigated cell lines (Fig. 9E).

### RNA sequencing of cell lines treated with tigecycline

**Fig. 10:**
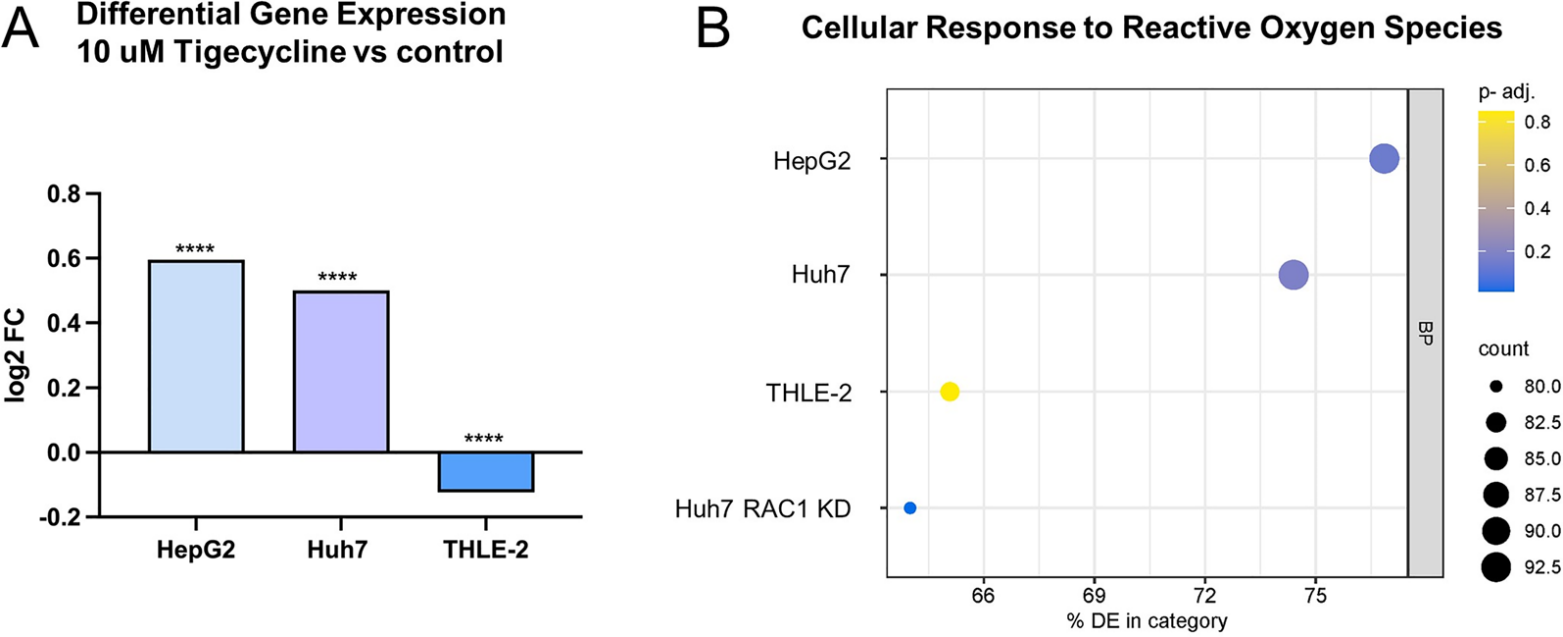
RNA expression after treatment with tigecycline. Differential Expression of RAC1 in HepG2, Huh7, and THLE-2 after 10 µM tigecycline for 48 h assessed by RNA bulk sequencing (**A**); Gene Ontology (GO) enrichment analysis for „Response to Reactive Oxygen Species “ (GO:0000302) in the category „Biological Processes “ displaying RNA sequencing results of HepG2, Huh7, THLE-2, and RAC1 knockdown Huh7 cells after treatment with 10 µM tigecycline for 48 h (**B**). *p < 0.05, **p < 0.01, ***p < 0.005, ****p < 0.001; ns = no significance compared with the control group

To further enlighten the relevance of RAC1 and the involved pathways, we performed RNA bulk sequencing after treatment with 10 µM tigecycline for 48 h. We found that RAC1 RNA expression in comparison to untreated cells was significantly increased in Huh7 and HepG2. In THLE-2, we found a decrease of RAC1 RNA in a significant but less pronounced amount (Fig. 10A).

Performing Gene Ontology (GO) enrichment analysis, major differences between the malignant cell lines Huh7 and HepG2 in comparison to the immortalized normal hepatocyte cell line THLE-2 were found focusing on the “Response to Reactive Oxygen Species“ (GO:0000302) in the category “Biological Processes “ (Fig. 10B). Also, RAC1 knockdown cells presented similar to the THLE-2 cells in this analysis. In detail, our findings revealed significant enrichment in HepG2 with 78.3% (adjusted p-value = 0.0118). Similarly, in Huh7, 77.2% of the genes were enriched (adjusted p-value = 0.0069). In contrast, THLE-2 displayed an enrichment of 69%, though it did not reach statistical significance (adjusted p-value of 0.1374). Furthermore, upon knocking down RAC1 in Huh7 (Huh7 RAC1 KD), the enrichment was reduced to 61.8%, yet this change remained statistically significant with an adjusted p-value of 0.0078.

Moreover, we analyzed differences in important cellular pathways based on our RNA sequencing date. Visualized in KEGG pathway, we found that tigecycline induced more changes in the malignant cell lines Huh7 and HepG2, but less influence on the signaling in THLE-2 cells (Additional file 1: Figure S7).

## Discussion

Treatment of HCC is predominantly surgical, with LT being the most favorable treatment option. Compared to LT, HCC resection does have much higher recurrence rates. However, especially in areas where donor organs are scarce, LR is the treatment of choice. To date, there are no established adjuvant therapeutic options to improve recurrence rates after LR to achieve comparable results to LT. Checkpoint inhibition yields hope and is currently tested in an adjuvant setting. Besides new immunotherapies, we suggest repurposing the antibiotic tigecycline as a novel and innovative perioperative and adjuvant treatment option.

Tigecycline exhibits antibacterial activity by binding to the 30S ribosomal subunit of bacteria and thereby blocking the interaction of aminoacyl-tRNA with the ribosomal A site [15]. Tigecycline has been shown to possess anticancer effects in studies of human acute myeloid leukemia, primarily by inhibiting mitochondrial translation [16]. Since then, an increasing number of anticancer studies on tigecycline have been published with pronounced effects in various solid tumors.

There are few studies evaluating the effects of tigecycline on HCC. In 2017, Tan J et al. found that tigecycline enhanced cisplatin activity in HCC by inducing mitochondrial dysfunction and oxidative damage [25]. A recent study has reported that tigecycline could be considered as a second-line treatment when HCC patients become resistant to Sorafenib [27]. To the best of our knowledge, no other basic science studies on tigecycline in HCC have been published, yet. Moreover, previous experiments never directly focused on potential target proteins of tigecycline in HCC and the effects on normal hepatocytes have never been investigated in detail.

Our study demonstrates that tigecycline inhibits the growth of different HCC cell lines (Huh7, HepG2, and Hep3B). Interestingly, there is a significant difference in the IC_50_ of the cell lines. HepG2 is more sensitive to tigecycline than Hep3B and Huh7. In particular, there is a big difference between the IC_50_ concentrations of HepG2 and Huh7 with 1.723 µM (IC50 HepG2) vs. 7.695 µM (IC_50_ Huh7). In both cell lines, the decrease in cell viability was due to inhibition of cell proliferation rather than cell death. To better simulate stem cell characteristics, we used sphere formation assays as a three-dimensional cell culture model. Here, the initial findings were confirmed. However, in contrast to conventional cell culture, we couldn’t detect a clear difference concerning sensitivity to tigecycline between the two malignant cell lines. In addition, we found that both migration and invasion were inhibited significantly by tigecycline. In search of mechanistic explanations, we examined changes in ROS production, OXPHOS, mitochondrial mass, and cell cycle phases in the cell lines after tigecycline treatment. To shed light on the lack of scientific knowledge regarding the effects of tigecycline on normal hepatocytes, we repeated all of the above-mentioned experiments with the immortalized normal hepatocyte cell line THLE-2. In addition, we used detailed bioinformatic analysis to search for potential target proteins of tigecycline in HCC. We identified RAC1 as a potential target gene and analyzed the changes in RAC1 in response to tigecycline treatment. Here, we also added RNA bulk sequencing upon tigecycline treatment to get further information.

The measured difference in sensitivity of Huh7 and HepG2 to tigecycline in two-dimensional cell culture model may be due to the fact that both cell lines display highly heterogeneous metabolic backgrounds. In 2018, Jian S et al. performed a principal component analysis of the expression profiles of 101 drug-metabolizing enzymes of Hep3B, HepG2 and Huh7 cell lines. Hep3B and Huh7 seemed very similar, while HepG2 differed significantly from them regarding expression patterns of drug-metabolizing enzymes [28]. A further discrepancy in the sensitivity to tigecycline is seen with the cell line THLE-2, an immortalized hepatocyte cell line of benign origin. We found that THLE-2 has a higher IC_50_ value (11.01 µM) than all tested HCC cell lines. This is an encouraging result and might suggest that tigecycline could particularly affect tumor cells with limited damage to normal hepatocytes if the dose is well chosen. However, the IC_50_ difference between the tested malignant cell lines and the immortalized normal hepatocyte THLE-2 cell line is small and the effects of tigecycline on cell viability and mitochondrial function not only occur in the malignant HCC cells. These effects can also be observed in a weakened manner in the THLE-2 cell line. Taking these aspects into consideration, it cannot be ruled out that this immortalized normal hepatocyte cell line model became adapted in culture towards a cancer-like behavior. However, it is promising, that the before mentioned IC_50_ concentrations (7.695 µM for Huh7, 1.723 µM for HepG2, and for 2.673 µM Hep3B) seem to be clinically achievable. When used as an anti-bacterial treatment, a concentration of about 0.5 µM can be reached in human blood by administering 50 mg tigecycline twice a day. At first glance, there seems to be a discrepancy between the experimental in vitro condition and concentrations of tigecycline that are usually needed for the treatment of bacterial infections. However, the following three points should help to remove those concerns. First, the dosages used for our in vitro setting are not directly comparable with tigecycline concentrations potentially needed for HCC treatment in humans. In the cell culture experiments presented here, we administer tigecycline merely once at the beginning of each experiment regardless of the time point, whereas clinical treatment of patients requires a dose twice a day. Second, higher doses of tigecycline may be used without major side effects. Theoretically, this allows an increase of the dose to achieve blood concentrations needed for oncological purposes. Third, we moreover suggest, that the tigecycline concentration in liver tissue is much higher than the systemic blood concentration based on data in colon tissue. In this report, Rubino et al. describe concentrations in colon tissue that were 173% higher than in corresponding blood samples [29].

In search of mechanistic explanations of tigecycline-induced antitumor effects, we further assessed cell metabolism and cell cycle after tigecycline treatment. Cell metabolism and cell cycle are prerequisites to tumor growth and their regulation can be an effective target for anticancer therapy [30, 31].

Regarding metabolism, we assessed mitochondrial OXPHOS and found that tigecycline significantly reduced OXPHOS and the expression of subunits of the respiratory chain complexes in Huh7 and HepG2 cells without reducing mitochondrial mass. Interestingly, both mtDNA- and nucDNA-encoded subunits of the respiratory chain complexes were reduced.

Also, in the immortalized normal hepatocytes THLE-2, OXPHOS was reduced. However, there was a difference between THLE-2 and HCC cell lines. Comparing all three treated cell lines with their respective untreated controls, THLE-2 showed very little changes in OXPHOS.

These effects on OXPHOS were initially measured after 48 h of tigecycline treatment. Also, the above-mentioned and discussed inhibitory effect on cell viability was only significant after 48 h of treatment. These results raised the question of cause and effect. One example of OXPHOS-mediated cytotoxicity is resveratrol. It is well documented that alterations in OXPHOS can modify the cytotoxicity of resveratrol, which is related to energy metabolism [32]. Resveratrol inhibits OXPHOS and in conditions with low energy accessibility, resveratrol can enhance ATP starvation to lethal levels. However, for tigecycline, we cannot exclude the possibility that increased cytotoxicity directly causes cell death and then OXPHOS decreases subsequently. To answer this question of cause and effect, we added a 6-h short-term tigecycline treatment of the three cell lines for measuring OXPHOS, because we had already found that treating Huh7 and HepG2 for only 24 h did not lead to significant inhibition of cell viability. Our experimental results demonstrate that the inhibitory effect of tigecycline on OXPHOS is reproducible and especially significant for HepG2 after 6 h of treatment whereas cell viability remained unchanged even at 24 h with comparable tigecycline concentrations as low as 10 µM. Taken together, we suggest that the effect on OXPHOS precedes the change in cell viability.

In our short-term experiments of tigecycline exposure for 6 h, the reduction of OCR was present in HepG2 but not in THLE-2. This is comparable with results obtained after 48 h of tigecycline exposure. Here, THLE-2 had the lowest OCR reduction upon tigecycline treatment. As mentioned before, this may be caused by different drug-metabolizing enzymes and hence, energy requirements. Tigecycline has a strong effect on the inhibition of OXPHOS metabolism within HepG2, resulting in a large reduction of energy production with low doses of tigecycline. Accordingly, cells that lack energy cannot perform DNA synthesis which eventually leads to growth inhibition or even death.

OXPHOS and cell metabolism are closely linked to the cell cycle. Numerous studies have demonstrated that tigecycline arrests the cell cycle in the G0/G1-phase in a variety of malignancies, including melanoma [33], glioma [22], neuroblastoma [34], oral squamous cell carcinoma [21], pancreatic ductal adenocarcinoma [35] and multiple myeloma [36]. These studies have generally associated a reduction in cyclin D1 and CDK family members (CDK2 and CDK4) with cell cycle arrest. Some studies also present a different view, with Bo Hu and Yue Guo in a study of ovarian cancer suggesting that tigecycline induces cell cycle arrest in the G2/M-phase rather than in the G0/G1-phase [37]. Interestingly, in HCC, our results support the idea that tigecycline leads to cell cycle arrest. We found that the proportion of S-phase cells decreased while the proportion of G0/G1 and G2/M-phase cells increased. We speculate that there is a close link to the lack of energy supply induced by tigecycline exposure leading to weakened mitochondrial OXPHOS. DNA synthesis occurs in the S-phase and both the initiation and completion of DNA synthesis and mitosis are energy-dependent and require OXPHOS in plant and animal cells [38, 39].

Our bioinformatic analysis revealed RAC1 as one potential target protein for tigecycline in HCC. We initially identified 11 potential genes that are relevant genes in HCC and code for potential target proteins of tigecycline. Among these 11 potential genes, only RAC1 differed significantly in expression compared to normal hepatocytes and survival prognosis analysis comparing RAC1 low and high expression in HCC. Subsequently, we chose RAC1 to continue our study. Bioinformatic results show that RAC1 is highly expressed in cancer and that high expression is associated with a worse prognosis. Numerous basic studies have also shown that reduced RAC1 expression was associated with the inhibition of HCC growth [40–43]. Interestingly, focusing on RAC1 in HCC, we have repeatedly confirmed increased expression of RAC1 in response to tigecycline exposure at both mRNA and protein levels. These findings were supported by our RNA bulk sequencing upon tigecycline exposure for the malignant cell lines Huh7 and HepG2. For the first time, we found that tigecycline increased the expression of RAC1 while inhibiting cell viability. This could be explained by a feedback loop of the cells to escape the treatment pressure induced by tigecycline. Also, it is possible, that only high RAC1 expression endure the tigecycline exposure.

After finding an increased RAC1 mRNA and protein expression upon tigecycline treatment in Huh7 and HepG2, we wondered how RAC1 might be affected in the non-malignant THLE-2 cells. Tigecycline treatment of THLE-2 led to an increased mRNA expression after 24 h of treatment without an equivalent translation into RAC1 protein expression. Interestingly, our RNA bulk sequencing upon 48 h of treatment with tigecycline could not confirm increased RAC1 RNA expression in THLE-2 which would be consistent with the non-existent translation into RAC1 protein in THLE-2. Such differences like RAC1 expression might therefore be responsible for the differential sensitivity to tigecycline of malignant and benign cells. Combined with the bioinformatic data, we believe that this may be due to the low protein expression of RAC1 in normal tissues or cells. We speculate that this phenomenon of increased mRNA without measurable protein expression after 24 h of treatment may be due to post-transcriptional modifications.

However, analyzing the total amount of RAC1 mRNA and protein expression is limited. In fact, RAC1 is distributed in different subcellular organelles, such as mitochondrial RAC1, nuclear RAC1, and plasma membrane RAC1 [44]. These different distributions of RAC1 may have different biological functions. Therefore, analyzing the total amount of RAC1 mRNA and protein may not be precise enough. Moreover, RAC1 can be modified by phosphorylation and the RAC1 activation, which means the GTP binding of RAC1, needs to be assessed in further studies.

A vast amount of literature suggests an inextricable relationship between RAC1 and ROS. Some articles suggest that changes in RAC1 are a prerequisite for changes in ROS [45–48], whereas other studies found that changes in ROS affect RAC1 activity [49, 50]. Regarding the effect of tigecycline, there is no data in the literature about the effect of tigecycline on RAC1 and not much data about the relationship between tigecycline and ROS. It is suggested that tigecycline increases the production of ROS [20, 25, 51]. Using ELISA and flow cytometry-based ROS measurements, we detected robustly increased amounts of ROS in Huh7 and HepG2 cells after tigecycline treatment after 48 h. Whether this increase of ROS influences cell viability or proliferation is unclear. ROS has been described as a double-edged sword in cell proliferation. It can either increase oxidative distress by activating the BAX/BCL-2 pathway and increasing the damage of DNA, proteins, and lipids, resulting in cell death and decreased proliferation. On the other hand, it can promote cell growth by increasing oxidative eustress through PI3K/AKT/mTOR, MAPK/ERK, and other pathways.

Interestingly, Gene Ontology enrichment analysis based on our RNA sequencing data revealed differences between the malignant cell lines Huh7 and HepG2 in comparison to THLE-2 focusing on the “Response to Reactive Oxygen Species”. Also, RAC1 knockdown cells presented similar to THLE-2.

## Conclusion

In this study, we were able to demonstrate an array of antitumor effects of tigecycline on HCC cells. First and foremost, the inhibition of mitochondrial OXPHOS and the G-protein RAC1 could be involved in mediating these effects. Tigecycline acts on malignant liver cells, while normal hepatocytes were less sensitive to tigecycline at clinically relevant concentrations. Based on these encouraging findings, we suggest further effort on exploring the effect of the antibiotic tigecycline on HCC in order to allow repurposing as a novel and innovative adjuvant or even perioperative therapeutic strategy in the setting of liver surgery for HCC in the future.

### Supplementary Information

**Additional file1**: **Figure S1.** Tigecycline induces cytostatic effect on HCC cells. **Figure S2.** Cell death after tigecycline treatment of HCC cells and normal hepatocytes. **Figure S3.** Cell death after tigecycline treatment of HCC cells and normal hepatocytes (Corresponding dot plots for Additional file 1: Figure S2). **Figure S4.** Wound healing and transwell assays with Huh7 and HepG2. **Figure S5.** Comparison of Huh7, HepG2 and THLE-2 cells. **Figure S6.** Bioinformatic analysis of potential tigecycline targets in HCC with RAC1 expression and survival analysis. **Figure S7.** RNA expression after treatment with tigecycline presented in KEGG pathways.

## Data Availability

The data that support the findings of this study are available within the article, its supplementary materials or are publicly available. Further inquiries can be directed to the corresponding author.

## Abbreviations

ANOVA: One/two-way analysis of variance
CTD: Comparative toxicogenomics database
cDNA: Complementary DNA
CV: Crystal violet
DCFH-DA: 2',7'-dichlorofluorescein diacetate
DFS: Disease free survival
EGA: European genome-phenome archive
ETC: Electron transport chain
FBS: Fetal bovine serum
GEO: The gene expression omnibus
GO: Gene ontology
HCC: Hepatocellular carcinoma
IC_50_: Half maximal inhibitory concentration
LIHC: Liver hepatocellular carcinoma
LR: Liver resection
LT: Liver transplantation
mtDNA: Mitochondrial DNA
MTT: 3-(4,5-Dimethylthiazol-2-yl)-2,5-diphenyltetrazolium bromide
NAO: Nonyl-acridine orange
ns: No significance
nucDNA: Nuclear DNA
OCR: Oxygen consumption rates
OS: Overall survival
PBS: Phosphate buffered saline
PFA: Paraformaldehyde
RFU: Relative fluorescent units
RIN: RNA integrity number
ROS: Reactive oxygen species
SD: Standard deviation
SFA: Sphere formation assay
TBST: Tris-buffered saline containing tween-20
TCGA: The cancer genome atlas
Tige: Tigecycline
