# Supplementary material for: Tigecycline causes loss of cell viability mediated by mitochondrial OXPHOS and RAC1 in hepatocellular carcinoma cells
Source: J Transl Med. 2023 Dec 2;21:876. doi: 10.1186/s12967-023-04615-4 (PMC10693093; doi:10.1186/s12967-023-04615-4)
Supplement: Supplementary file 1 — Additional file1: Figure S1. Tigecycline induces cytostatic effect on HCC cells. Figure S2. Cell death after tigecycline treatment of HCC cells and normal hepatocytes. Figure S3. Cell death after tigecycline treatment of HCC cells and normal hepatocytes (Corresponding dot plots for Additional file 1: Figure S2). Figure S4. Wound healing and transwell assays with Huh7 and HepG2. Figure S5. Comparison of Huh7, HepG2 and THLE-2 cells. Figure S6. Bioinformatic analysis of potential tigecycline targets in HCC with RAC1 expression and survival analysis. Figure S7. RNA expression after treatment with tigecycline presented in KEGG pathways. [file 12967_2023_4615_MOESM1_ESM.docx]

**Supplementary Files**

**Supplementary Figures**


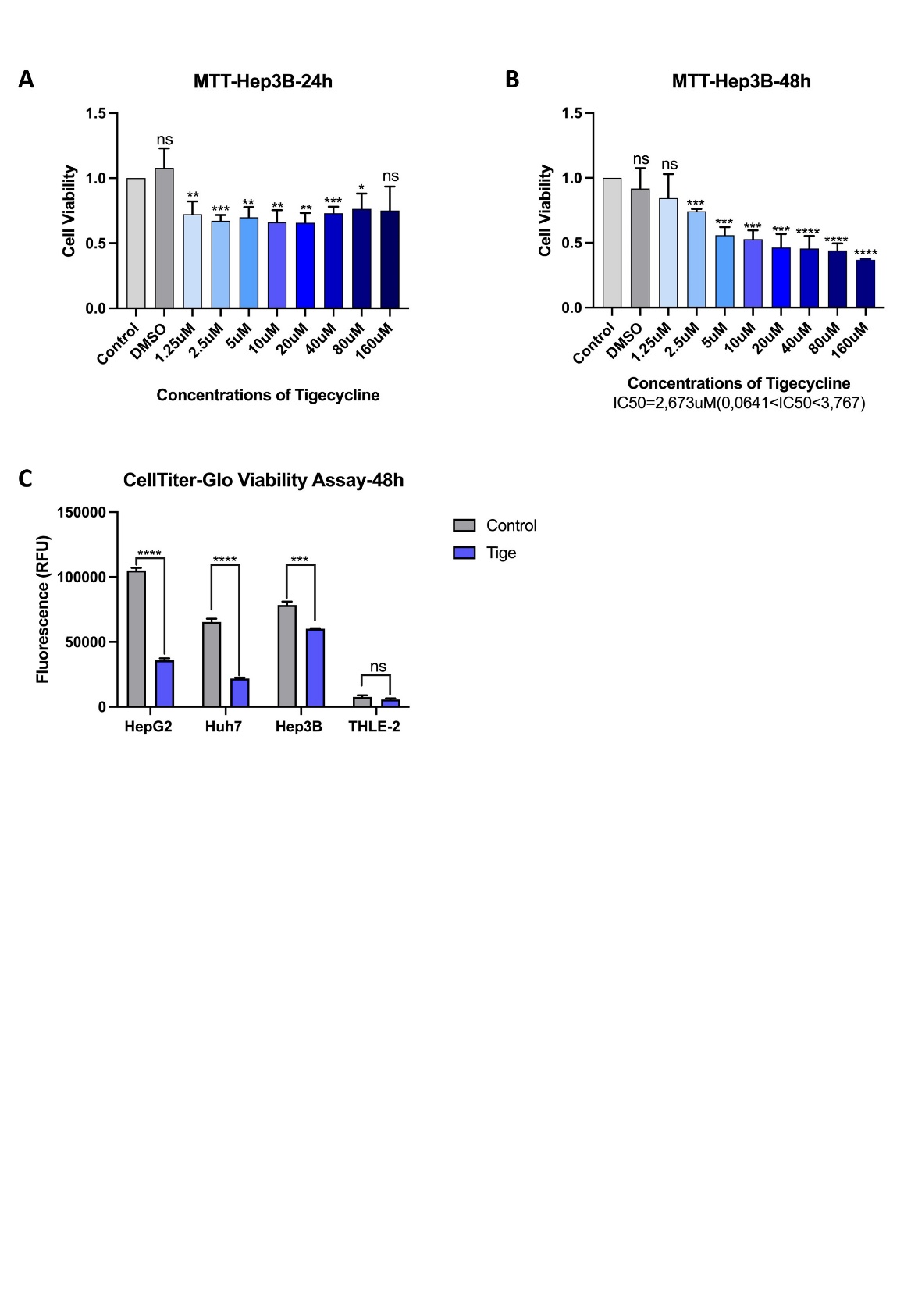


**Supplementary Figure 1:** Tigecycline induces cytostatic effect on HCC cells.

MTT based cell viability of Hep3B treated with increasing concentrations of tigecycline for 24 hours (A) and 48 hours (B). Cell viability assessed with CellTiter-Glo Viability Assay after 48 hours of treatment with 10 µM tigecycline for all used cell lines (C).

Bar graphs represent the mean ± SD; *p<0.05, **p<0.01, ***p<0.005, ****p<0.001; ns = no significance compared with the control group (grey bar graphs).

MTT, 3-(4,5-Dimethylthiazol-2-yl)-2,5-Diphenyltetrazolium Bromide; IC50, half maximal inhibitory concentration.


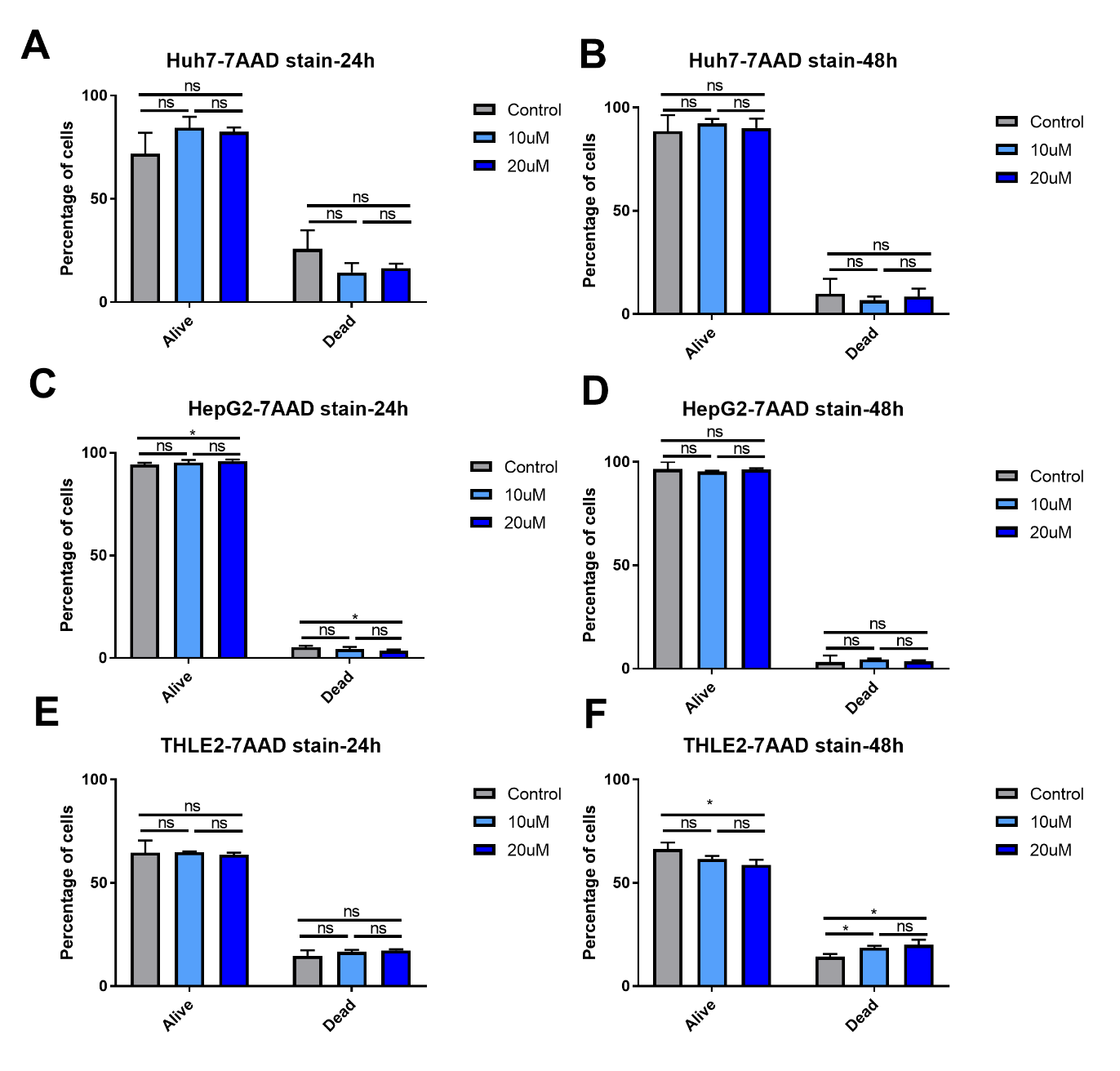


**Supplementary Figure 2:** Cell death after tigecycline treatment of HCC cells and normal hepatocytes.

Percentage of alive and dead cells after treatment with 10 and 20 µM tigecycline assessed by flow cytometry and 7-AAD viability staining in Huh7 (A and B), HepG2 (C and D) and THLE-2 (E and F) cells.

Bar graphs represent the mean ± SD; *p<0.05, **p<0.01, ***p<0.005, ****p<0.001; ns = no significance compared with the control group (grey bar graphs).


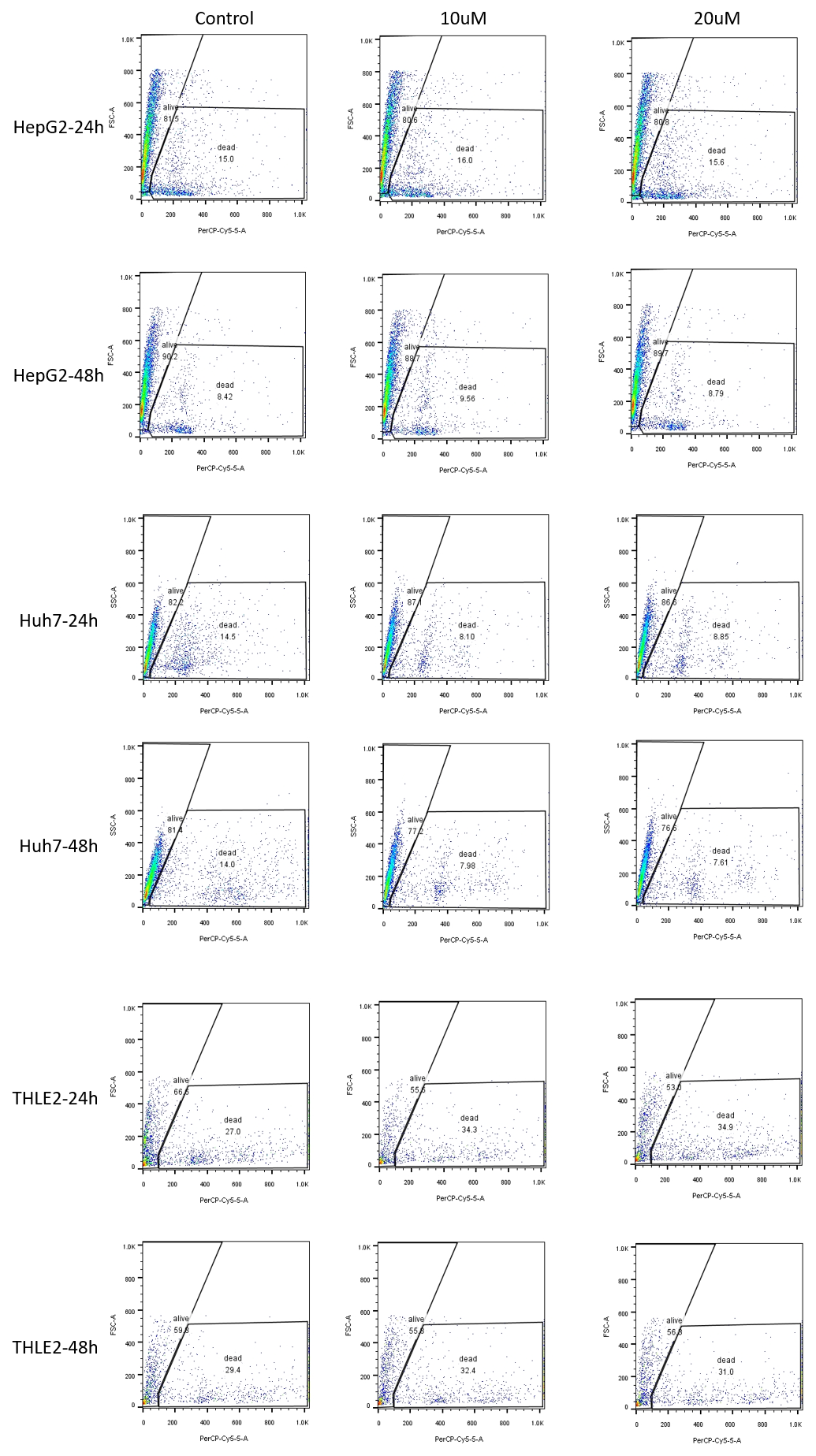


**Supplementary Figure 3:** Cell death after tigecycline treatment of HCC cells and normal hepatocytes (Corresponding dot plots for Supplementary Figure 2).

**
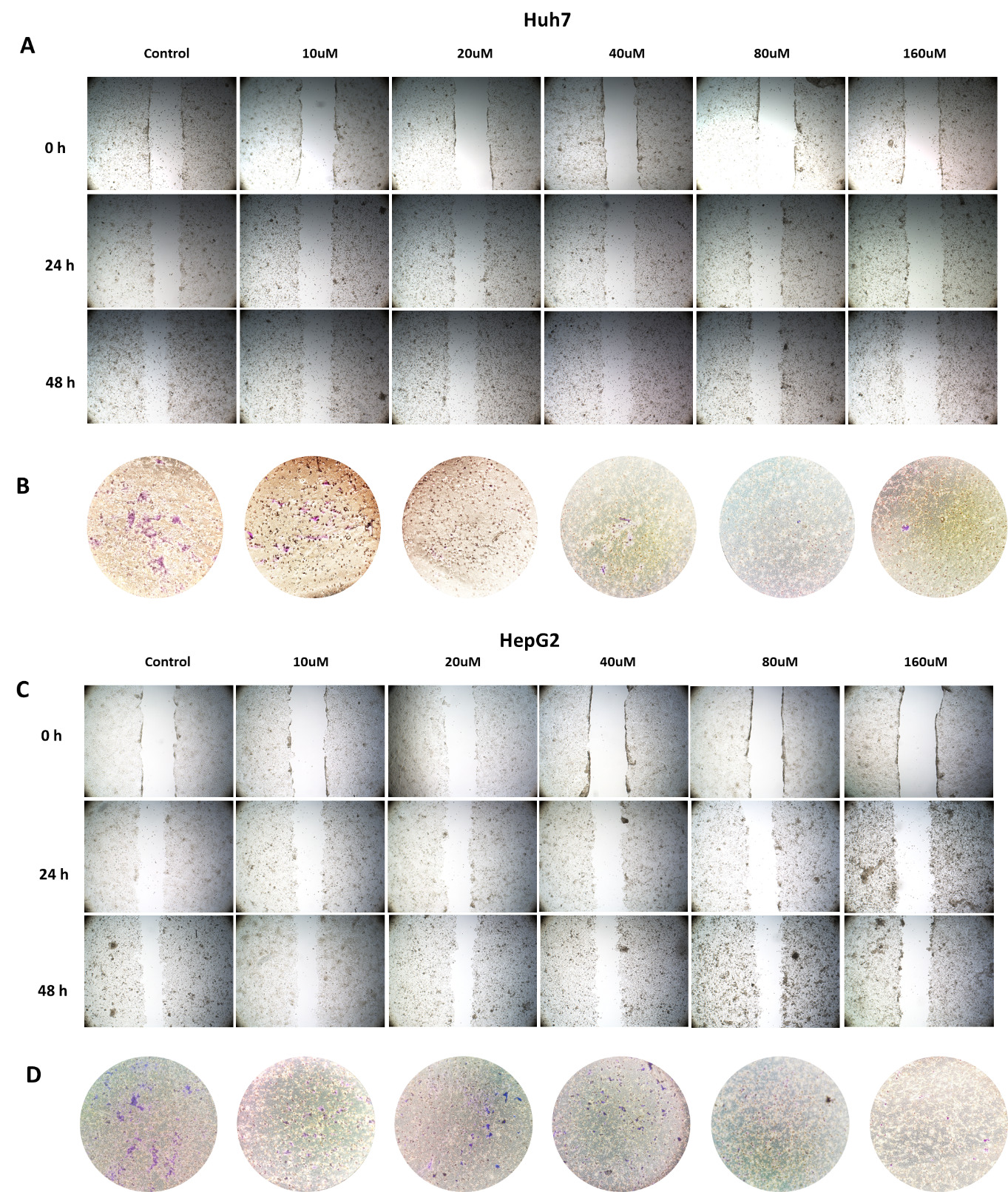
**

**Supplementary Figure 4:** Wound healing and transwell assays with Huh7 and HepG2.

Microscope images at different time points of wound healing assays with Huh7 (A) and HepG2 (C) with increasing tigecycline concentrations (magnification 50x); Microscope images at different time points of transwell assays with Huh7 (B) and HepG2 (D) with increasing tigecycline concentrations (magnification 100x).

**
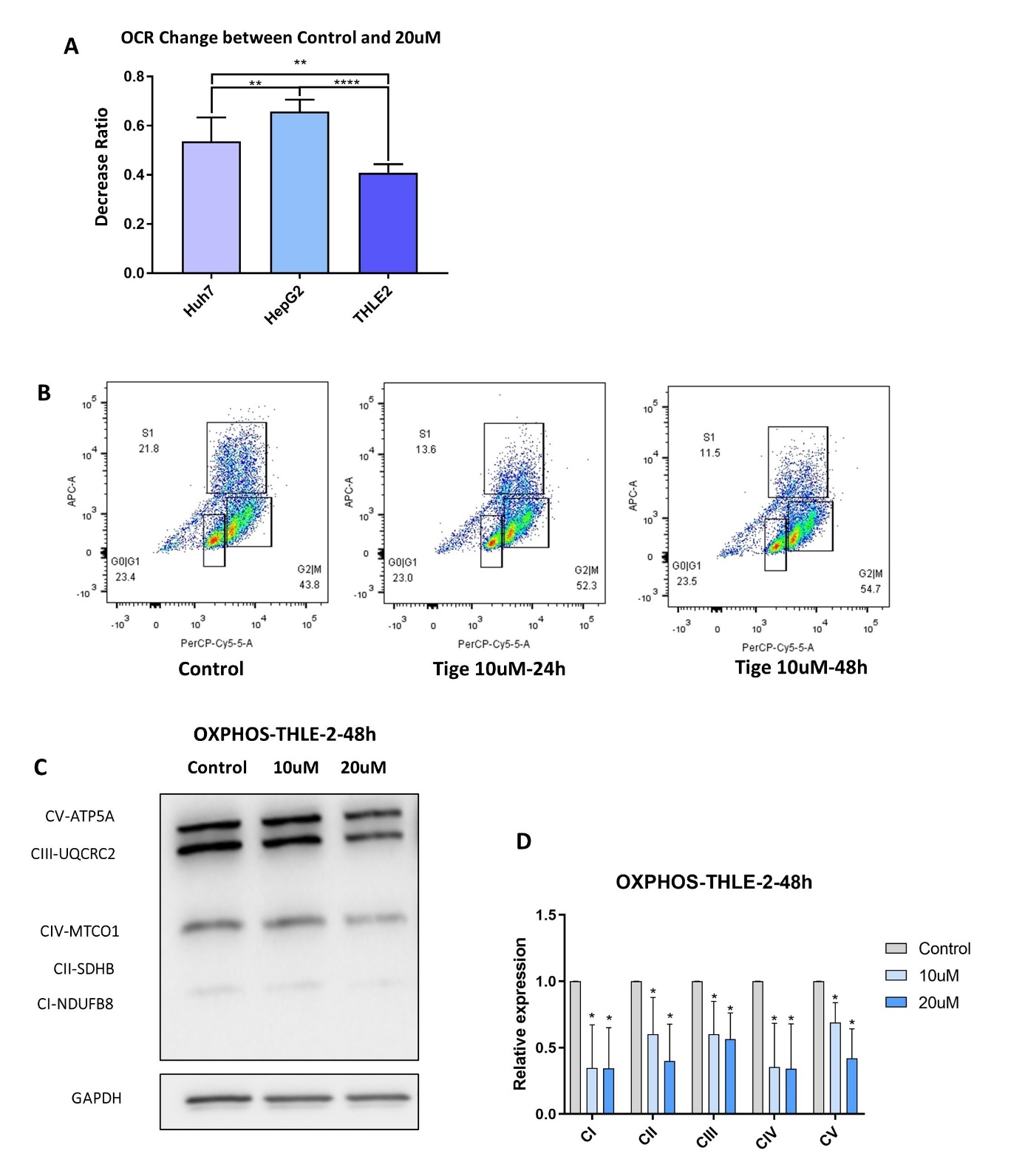
**

**Supplementary Figure 5:** Comparison of Huh7, HepG2 and THLE-2 cells.

Comparison of the relative reduction of OCR in basal respiration after treatment with 20 µM tigecycline for 48 hours compared to the respective control group of Huh7, HepG2 and THLE-2 cells (A); Flow cytometry dot plots showing the changes in cell cycle of THLE-2 after 10 µM tigecycline treatment for 24 and 48 hours (B); Changes in the protein expression of respiratory chain subunits in THLE-2 after tigecycline treatment (C); Relative protein expression of respiratory chain subunits after tigecycline treatment of THLE-2 for 48 hours (D).

Bar graphs represent the mean ± SD; *p<0.05, **p<0.01, ***p<0.005, ****p<0.001; ns = no significance compared with the control group (grey bar graphs).

CI, complex I; CII, complex II; CIII, complex III; CIV, complex IV; CV, complex V.


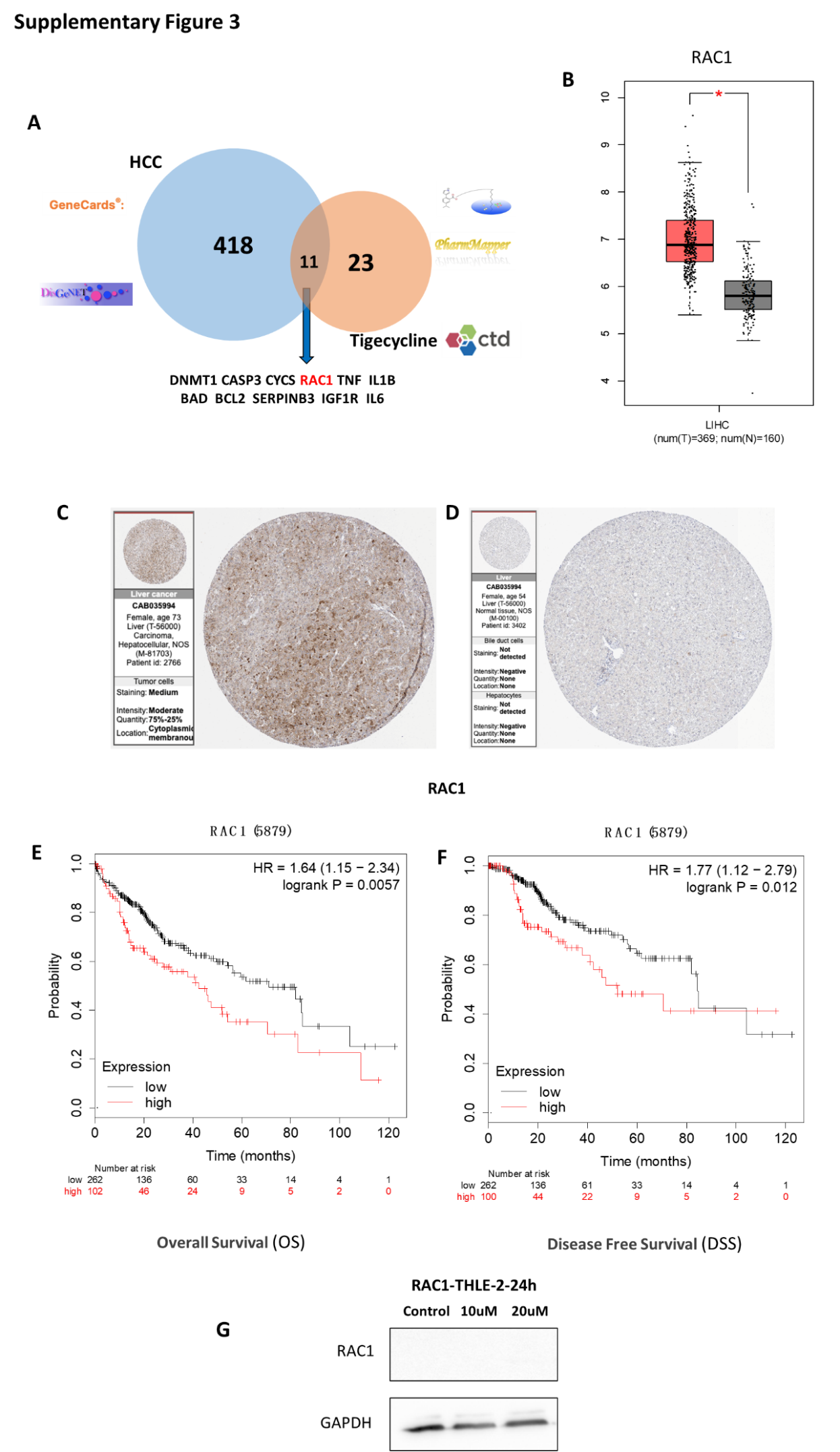


**Supplementary Figure 6:** Bioinformatic analysis of potential tigecycline targets in HCC with RAC1 expression and survival analysis.

429 potential genes related to HCC derived from the GeneCards and DisNET and 34 potential target genes for tigecycline from Pharmmaper and Comparative Toxicogenomics Database were identified. The intersection reveals 11 relevant genes (A); Expression of RAC1 in HCC compared to normal tissue with data is derived from Gepia (http://gepia.cancer-pku.cn/) (B); Protein expression of RAC1 in HCC (C) and normal tissue (D) with data derived from The Human Protein Atlas (https://www.proteinatlas.org/); Survival analysis of OS (E) and DFS (F) of patients with different expression of RAC1 with data derived from Kaplan-Meier plotter based on data from GEO, EGA, and TCGA (https://kmplot.com/analysis/index.php?p=service&cancer=liver_rnaseq). RAC1 protein expression in THLE-2 (G).

LIHC, liver hepatocellular carcinoma; OS, overall survival; DFS, disease free survival


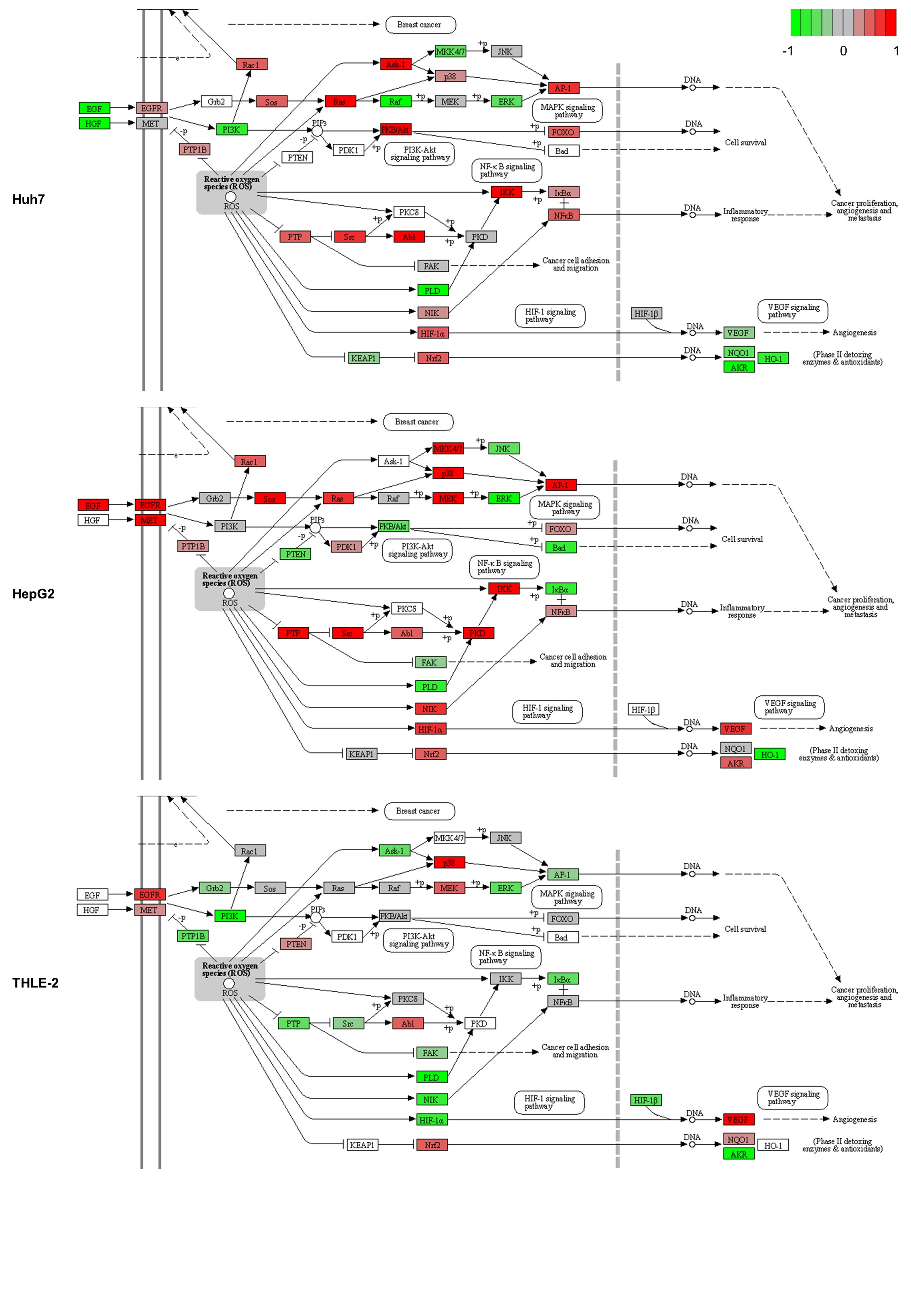


**Supplementary Figure 7:** RNA expression after treatment with tigecycline presented in KEGG pathways.

Comparison of RNA expression of the three cell lines Huh7, HepG2, and THLE-2 after 48 hours of treatment with 10 µM tigecycline compared to their untreated controls.

From green to red: Green indicates lower RNA expression and red means higher RNA expression compared to the untreated control.
